# Corded Ware cultural complexity uncovered using genomic and isotopic analysis from south-eastern Poland

**DOI:** 10.1038/s41598-020-63138-w

**Published:** 2020-04-14

**Authors:** Anna Linderholm, Gülşah Merve Kılınç, Anita Szczepanek, Piotr Włodarczak, Paweł Jarosz, Zdzislaw Belka, Jolanta Dopieralska, Karolina Werens, Jacek Górski, Mirosław Mazurek, Monika Hozer, Małgorzata Rybicka, Mikołaj Ostrowski, Jolanta Bagińska, Wiesław Koman, Ricardo Rodríguez-Varela, Jan Storå, Anders Götherström, Maja Krzewińska

**Affiliations:** 1 The BiG lab (Bioarchaeology and Genomics Laboratory), Department of Anthropology, Texas A&M University, College Station, USA; 2 Centre for Palaeogenetics, 10691 Stockholm, Sweden; 3 Department of Bioinformatics, Graduate School of Health Sciences, Hacettepe University, 06100 Ankara, Turkey; 4 Institute of Archaeology and Ethnology, Polish Academy of Sciences, Cracow, Poland; 5 Department of Anatomy, Jagiellonian University, Medical College, Cracow, Poland; 6 Isotope Laboratory, Adam Mickiewicz University, Poznań, Poland; 7 Poznań Science and Technology Park, Poznań, Poland; 8 School of Archaeology 34-36 Beaumont Street, Oxford, OX1 2PG United Kingdom; 9 Department of History and Cultural Heritage, University of Pope Jan Paweł II, Cracow, Poland; 10 Archaeological Studio Mirosław Mazurek, Rzeszów, Poland; 11 Łańcut Castle Museum, Łańcut, Poland; 12 Institute of Archaeology, University of Rzeszów, Rzeszów, Poland; 13 Archaeological Society “ARGO”, Cracow, Poland; 14 Regional Museum in Tomaszów Lubelski, Tomaszów Lubelski, Poland; 15 Provincial Office for the Protection of Cultural Heritage, Zamość, Poland; 16 Osteoarchaeological Research Laboratory, Department of Archaeology and Classical Studies, Stockholm University, Stockholm, Sweden

**Keywords:** Archaeology, Archaeology, Population genetics, Population genetics

## Abstract

During the Final Eneolithic the Corded Ware Complex (CWC) emerges, chiefly identified by its specific burial rites. This complex spanned most of central Europe and exhibits demographic and cultural associations to the Yamnaya culture. To study the genetic structure and kin relations in CWC communities, we sequenced the genomes of 19 individuals located in the heartland of the CWC complex region, south-eastern Poland. Whole genome sequence and strontium isotope data allowed us to investigate genetic ancestry, admixture, kinship and mobility. The analysis showed a unique pattern, not detected in other parts of Poland; maternally the individuals are linked to earlier Neolithic lineages, whereas on the paternal side a Steppe ancestry is clearly visible. We identified three cases of kinship. Of these two were between individuals buried in double graves. Interestingly, we identified kinship between a local and a non-local individual thus discovering a novel, previously unknown burial custom.

## Introduction

The Neolithic Stone Age of continental Europe saw important demographic changes and population events which in recent years have been demonstrated by numerous archaeogenomic studies^1–6^. The genetic ancestry, affinity and admixture processes between human groups have been traced by analyses of individuals from different time periods and geographical contexts which contextually are associated to cultural complexes with differing lifeways, burial customs and material culture expressions. The identification of the demographic event and successive development associated with the Neolithization of Europe demonstrated the importance of mobility and migration in the process of population turnover and transition towards changed lifeways (e.g.^7–9^). The process took different paths in different areas of Europe which is evident in the admixture patterns between the hunter-gatherer (HG) and the farming groups but also between farming and pastoralist groups, depending on the time period. The significant transitional process around 3000 cal BCE and the appearance of the (Pontic Steppe) Yamnaya cultural complex in eastern Europe is associated with a wave of migration from East that had marked impact on the demographic (a Steppe ancestry component), cultural and social as well as linguistic development in the third Millennium BCE^1,10^.

The appearance of the Yamnaya (around 3100-3000 BCE) complex in southern Europe roughly coincides with the appearance of the Corded Ware complex (CWC) (around 2900-2800 BCE) further to the North. Earlier archaeogenomic analyses have shown that the CWC individuals, exhibit Steppe ancestry^1,10–12^. Contemporaneous individuals analysed from this region (i.e. central Europe) have however shown a varying degree of Steppe ancestry^1,10^. This component has not been demonstrated among individuals associated with the Globular Amphora culture^11,13,14^. Although our knowledge about the demographic prehistory in continental Europe has increased immensely over the last five years^1–6^ the regional resolution of the events is insufficient and, thus, our understanding of the social processes is still lacking. The population dynamics and interpretations of the archaeogenomic data of the Yamnaya and the Corded Ware complexes and their relationships have been intensely debated and reviewed (see e.g.^15–18^).

Around 5400 BCE farming was introduced in central Poland which coincided with the appearance of the Linear Pottery culture (LBK) complex marking the transition to the Early Neolithic period. The HG population living in a neighbourhood of the LBK area upheld their lifeways and for almost a millennium the dispersal of the Neolithic lifeways halted. However, there were evidently contacts between the farming and HG groups seen in admixture patterns^11^. These relations are clearly visible in the Brześć Kujawski group (of the Lengyel complex) found in the region of Kuyavia which for a long time was the northern limit of the (post LBK) farming communities^11,19^.

During the next millennium, from around 4000 BCE, i.e. the Middle Eneolithic, the Funnel Beaker culture (TRB) emerges and the Eneolithic lifeways dispersed to the Northern parts of central Europe, including parts of Scandinavia, and now to most of the Polish area. Individuals from TRB contexts in Poland exhibit admixture with the HG-populations which were mostly linked to the west European HG group (WHG) (e.g.^11^). Around 3100 BCE the TRB manifestations in Poland decline with the appearance of the Baden culture and the Globular Amphora culture (GAC, c.3400/3100-c.2800 BCE) which replaced the earlier TRB complex in many areas of Poland. The GAC was a rather short-lived phenomenon and after 2800 BCE the Corded Ware Complex (CWC) manifestations became dominant, and continued for another 500 years before that complex disappears^11,20^.

Individuals of the GAC and CWC have very different ancestry. Individuals from CWC contexts in Poland exhibit a marked input from the eastern Yamnaya Steppe pastoralist whereas the individuals of the GAC complex hardly exhibit any Steppe ancestry^11,13,19^. Around 2400 BCE the Bell Beaker culture (BBC) complex enters this region, from the southwest, and yet another major genetic component is added to the local population^5,21^. In south-eastern Poland, the BBC disappears around 2200 BCE^22,23^. The development in Poland clearly shows the extent of admixture that has taken place in continental Europe and the importance of mobility in the demographic and social developments^10,11^. (Supplementary Information - The Corded Ware and the Bell Beaker societies in the Małopolska uplands).

By identifying mtDNA, Y-chromosomal DNA and nuclear DNA; ancestry, origin and admixture can be ascertained^24–27^. It has been shown that Yamnaya pastoralists contributed Y chromosome R1a and R1b haplogroups to continental Europe almost entirely replacing the previously wide-spread G2a haplogroup^1,2^. The mtDNA contribution of Yamnaya to CWC individuals is associated with the appearance of U2 and W haplogroups. This large-scale contribution, close to complete replacement is very evident among CWC individuals that have retained very little genetic ancestry from the Mesolithic HG and Early Neolithic farmer groups. The study of individuals associated with the CWC complex detected ancestry and admixture patterns with the HG groups, individuals of the Steppe culture groups such as Afanasievo and Yamnaya complexes. Moving forward in time evidence of admixture with individuals of the succeeding BBC complex appears, suggesting that this region of prehistoric Europe was an important social arena and melting pot of human genetics from both the east and the west^5^. Archaeogenomic analyses have the best possibilities to decipher demographic processes and the individuals associated to the CWC complex provide important clues to the development in the 3^rd^ Millennium BCE.

One of the most characteristic features of the CWC complex are the funeral rituals that quickly spread over a large part of the Central Europe^28^. Attempts have been made to identify and understand the migration patterns within this vast cultural complex, and whether there were regional subgroups or other kinds of subdivisions^11,12,20,29^. To identify and to understand how different subgroups of the Corded Ware complex interacted with each other and the surrounding populations, an in-depth analysis of individuals representing this cultural complex during a highly important period in prehistory (2800-2300 BCE) is of great importance^29^. The present study goes deeper by also examining kinship of the individuals, which will aid the understanding of the intricate networks and social structures of the CWC subgroups^28^. By further investigating the genomic signatures among the regional CWC population in south-eastern Poland, a more complete yet complex image will emerge with several admixture events from different cultural groups.

## Materials

**Figure 1:**
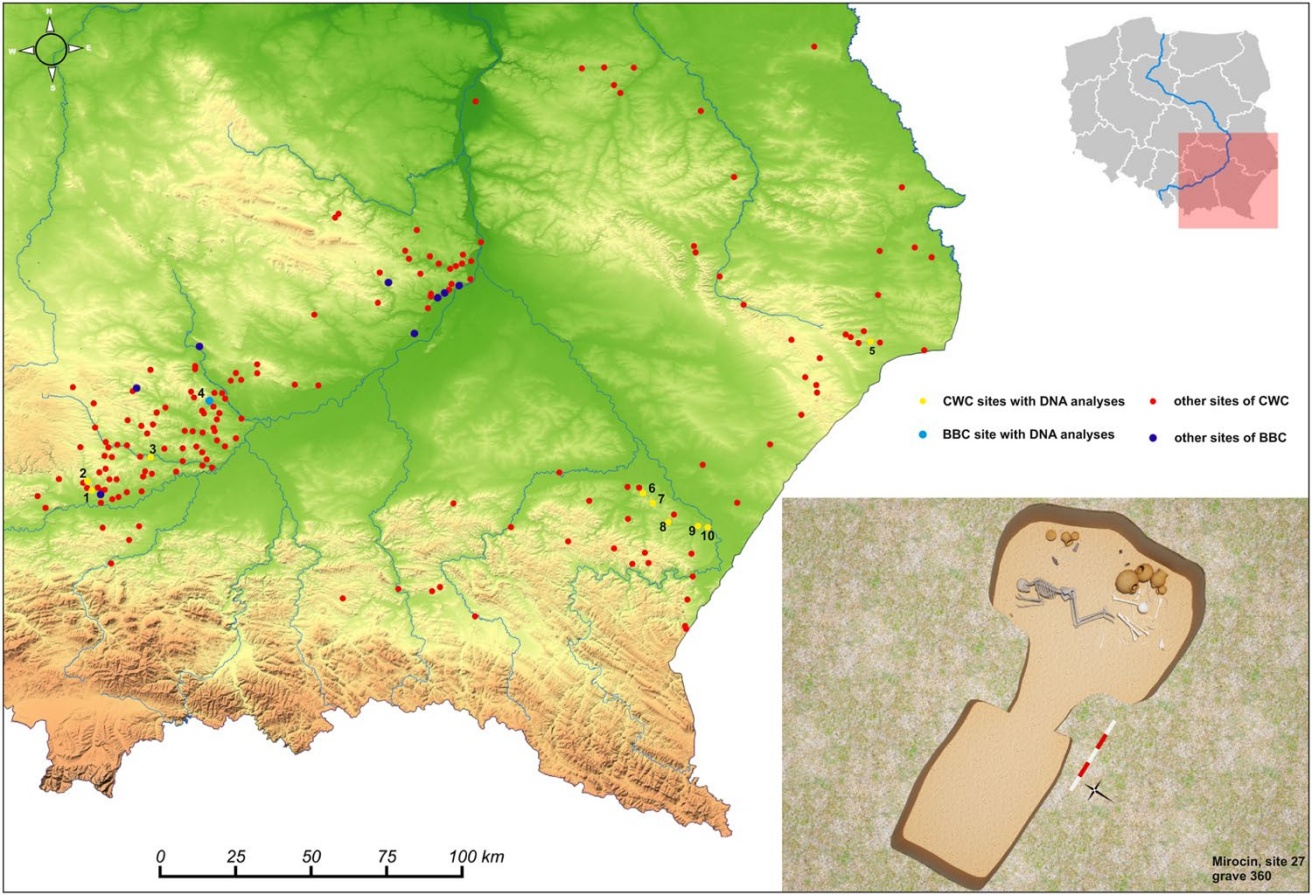
The relief map of south-eastern Poland with marked location of archaeological sites of the CWC and BBC cultures. 1 – Kraków-Mistrzejowice, 2 – Bosutów, 3 – Proszowice, 4 – Pełczyska, 5 – Łubcze, 6 – Mirocin, 7 – Szczytna, 8 – Chłopice, 9 – Skołoszów, 10 – Święte. Reconstruction of niche grave 360 from Mirocin, site 27 (drawn by K. Rosińska-Balik).

**Figure 2:**
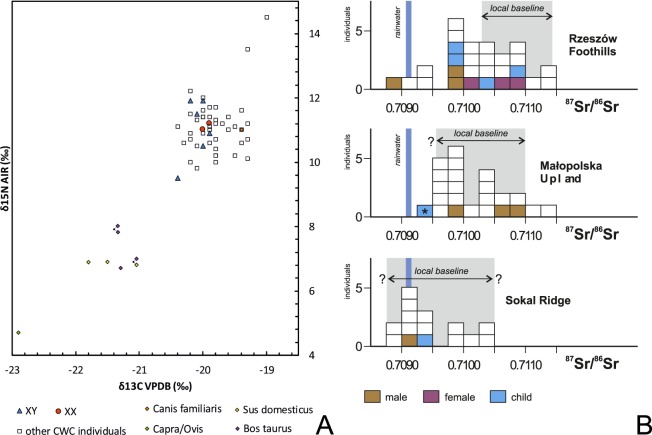
(**A**) Variation in carbon and nitrogen isotope compositions of the bone collagen of humans and animals of the Corded Ware Complex from south-eastern Poland. Individuals with DNA profiles (XX, XY) are indicated. (**B**) Strontium isotope composition (^87^Sr/^86^Sr) of human enamel of the CWC populations from the Rzeszów Foothills, Małopolska Upland and the Sokal Ridge. A child from the BBC grave at Pełczyska is asterisked. Individuals investigated during the present study are coloured. Other isotopic data and local baselines (in grey) are from Belka *et al*.^41^, Szczepanek *et al*.^29^ and results of unpublished investigations of the authors. The Sr isotope composition of rainwater is indicated.

Samples of the petrous portion of the temporal bone were taken from 50 individuals representing the Corded Ware Complex (CWC) and 3 individuals representing the Bell Beaker culture (BBC) buried in graves in south-eastern Poland. We have obtained genetic data from 19 individuals (16 of CWC and 3 of BBC). All examined individuals come from three geographical regions (Fig. 1; Table 1; Table S1): the Rzeszów Foothills (part of the Subcarpathian Region; sites of Szczytna, Chłopice, Mirocin and Święte), the Małopolska Upland (Mistrzejowice, Proszowice, Bosutów, Pełczyska) and the Sokal Ridge (the western part of Volhynian Upland – site of Łubcze) (Supplementary Information – Materials; Figs. S1–S13). All burials are of similar type exhibiting the same funeral rite with some differences concerning grave goods and their radiocarbon dates coincide (Table 1; Supplementary Information - 14 C Dating; Table S2, Fig. S15). The Strontium isotope (Sr) analyses included human enamel samples obtained from 16 individuals who were selected for sampling based on preserved dental enamel and availability of contextual information. Among them were females and males, predominantly of adult and mature individuals, and also children. Enamel was taken from first molars (M1) whenever possible and occasionally from first premolars (P1) (Supplementary Information – Strontium Isotopes; Table S4, Fig. 2). Moreover carbon (δ^13^C_coll_) and nitrogen (δ^15^N_coll_) stable isotope analyses were performed for 8 individuals (Supplementary Information – Stable isotopes – diet; Table S5, Fig. 2A). We investigated patterns in the genetic variation in relation to geographical subgroupings based on archaeological information but also the genetic data in relation to the other results obtained through the stable isotope (Sr) analyses and radiocarbon dating. The groups are listed in Table 1. In short, we first divided individuals based on geographical and archaeological context into four groups (Groups I-IV), and in addition introduce a division based on results from the strontium analyses (Groups Ia, IVa and V): For more details regarding tested groupings and obtained results, see section *Grouping individuals according to their provenance (Sr isotopes)*.

**Table 1:** Basic sample info, stats, division, dating, isotopes. Cells highlighted in grey represent non-local individuals from Rzeszów Foothills which constitute Group V.

Site/feature	Atlas ID	Site grouping	Sr Grouping	Genome coverage	Mol. Sex	MtDNA Hg	Y chromosome Hg	Lab no.	14 C (BP)
Szczytna 6/84	pcw110	Rzeszow Foothills (CWC) RFCWC **Group I**	Rzeszów Foothills (CWC) **Group Ia**	0.04	XY	T1	R1b1a1a2		
Chłopice 26/11	pcw212			0.12	XX	H2a2b	—	Poz-90881	3985 ± 35
	pcw211			0.96	XX	H2a2b	—		
Święte 15/408b	pcw062			0.02	XX	HV2a	—		
Mirocin 27/360	pcw160		Sokal Ridge & nonlocals from the Rzeszów Foothills (CWC) **Group IVa**	0.04	XY	I2	NA	Poz-54043	3870 ± 35
Święte20/40 A	pcw040			2.67	XY	U5b2b1a1	R1b1a1a2	Poz-90777	3950 ± 35
Święte 20/43/I	pcw041			0.31	XY	J1c2c	R1b1a1a2	Poz-90778	3950 ± 35
Święte 15/408a	pcw061			0.77	XX	H7a	—	Poz-90780	3890 ± 35
Święte 11/876	pcw070			0.79	XY	I2	R1b1a1a2	Poz-90875	3890 ± 35
Skołoszów 7/256	pcw191			0.09	XX	T2b11	—	Poz-55335	3830 ± 35
Łubcze 2/2	pcw350	Sokal Ridge (CWC) SRCWC **Group IV**		0.23	XY	T1a1	R1b1a1a2	Poz-90898	3865 ± 35*
Łubcze 25/3	pcw361			3.28	XY	K1a4b	R1b1a1a2a1a		
	pcw362			5.47	XY	U5a1a2a	R1b1a1a2a1a	Poz-90899	3875 ± 35^*^
Mistrzejowice 85/1311	pcw250	Malopolska Upland (CWC) MUCWC **Group II**		0.08	XY	T1a1	NA		
Proszowice 1/2	pcw420			0.03	XY	L3c’d	R		
Bosutów/1	pcw430			0.13	XY	U4b1b2	R		
Pełczyska 6/12	pcw260	Malopolska Upland (BBC) MUBBC **Group III**		1.59	XX	T2b	—	Poz-34734	3830 ± 35
Pełczyska 6/13	pcw270			0.08	XX	H	—		
Pełczyska 6/25	pcw280			0.41	XX	U5a1	—		

*unpublished earlier.

## Results

We collected bone samples from 53 individuals and produced low to medium coverage whole genome sequence data with coverages ranging between 0.02x to 5.4x for 19 Final Eneolithic/Bronze Age individuals from the ten sites in southern Poland. Ten of 19 sequenced individuals were radiocarbon dated from 3985 ± 35 to 3830 ± 35 BP (Fig. S14, Tables S2, S7). Obtained genetic sequences displayed cytosine deamination patterns characteristic of ancient DNA (Fig. S17)^8,30–32^. Mitochondrial (mtDNA) DNA-based contamination levels were calculated based on private polymorphism characterization (13 individuals with sufficient mtDNA coverage)^33^, and sequence mapping likelihood estimation^34^. The obtained contamination estimates varied between 0–3.6% (95% CI of 0–7.3%) and all individuals carried sequences with >90% (>97% in 14 individuals) probability of being authentic (Table S10). Thus, the obtained genomic data were deemed authentic and all sequenced individuals were included in population analyses.

All Eneolithic individuals from Poland carried mtDNA lineages of European or West Eurasian origin^35^ including H (including H2 and H7), HV, I2, J1, K1, T1, T2, U4, and U5 (Table S8). Individuals from Pełczyska exhibited the same mtDNA haplogroups identified at other CWC sites. In contrast to individuals of the Yamnaya complex the CWC and BBC individuals from southern Poland carried I2 and J1 lineages, but lacked the mtDNA haplogroups W and U2, often found in Yamnaya individuals^1,10,36^.

Molecular sex was assigned in all 19 individuals^37^ of which ten were sub-adults and therefore lacking prior osteological sex assessments. Eight individuals were female (XX) and 11 individuals were male (XY). The Y chromosome haplogroup was assigned in nine males of which all belonged to macrohaplogroup ‘R’ (Table S9). In seven individuals the Y chromosome haplogroup was further narrowed down to lineage R1b-M269 or R-L11 characteristic of Yamnaya and Bell Beaker individuals^5,10^ and particularly widespread throughout Eurasia since the Bronze Age^38^.

In order to investigate mutual relations between individuals we employed conditional nucleotide diversity estimates which is calculated between all pairs of individuals investigated in this study^7^ (Table S11). Here an average number of mismatches between pairs of individuals was estimated based on sites in Human Origins dataset and in Yoruban individuals from 1000 Genomes Project (Table S11)^39^. The results did not reveal strong structuring between sites but highlighted closer relationships between a number of individuals (pcw040-pcw041, pcw061-pcw062 and pcw211-pcw212). Additionally, reduced conditional diversity was observed between an individual from Proszowice (pcw420) and individuals from Święte (pcw062, pcw110) and Skołoszów (pcw191).

### Principal Component Analysis (PCA)

**Figure 3:**
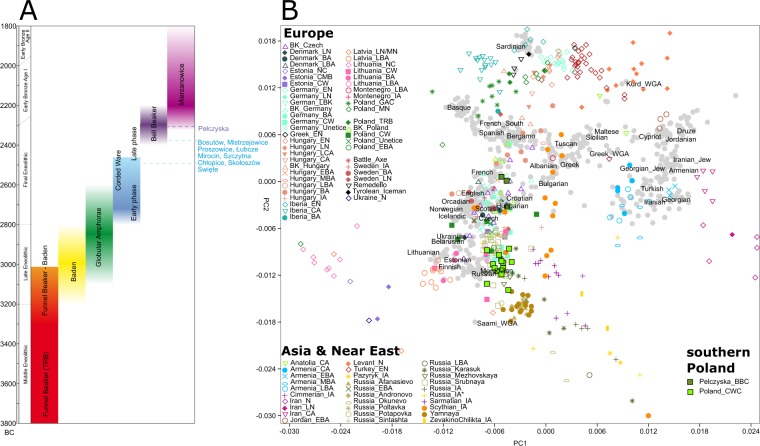
Cultural timeline approximately visualising occurrence of different cultural complexes and their dates with the southern Poland samples marked on the left (**A**); PCA visualising genetic variation of modern European populations marked as grey dots while ancient samples (spanning Neolithic to Iron Age) are following key presented in the legends (**B**).

In order to investigate and visualize genetic relations between our Polish Corded Ware individuals and both present-day and ancient populations we performed PCA on the autosomal genomic data (Figs. 3B, S21, S22). The PCA suggests that (a) despite geographical proximity there is a distinct genetic separation between CWC and BBC individuals from Southern Poland. (b) the genetic variation of CWC individuals from southern Poland overlaps with the majority of the published CWC individuals from Germany while the eight published CWC individuals from Poland^10,11^ show a closer similarity to BBC representatives (Fig. S20) (c) the genetic variation of BBC individuals from southern Poland overlaps with the broad variation of BBC individuals from Central Europe (Czech Republic, Germany, Poland and Hungary) (Fig. S21). Based on the archaeological, geographical and genetic results we further ran tests according to cultural division (two groups: CWC and BBC) and geographical distribution of sites (four groups: Group I-IV) (Table 1).

### Admixture

**Figure 4:**
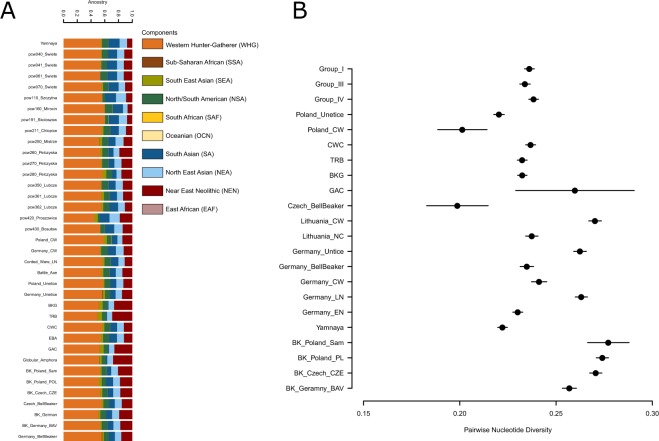
A pruned visualisation of the admixture run of the whole dataset at K = 10 (Fig. S23). The bar plot shows newly published data from Poland as well as previously published individuals from Poland, Yamnaya, Battle Axe cultures and Corded Ware, Bell Beaker individuals from Germany and Czech Republic (**A**). Conditional nucleotide diversity in Groups I, II and IV compared to diversity estimates from other closely related population groups (**B**).

In order to trace ancestral whgs of the samples we performed ADMIXTURE analyses between 2 K and 10 K using 10 replicates per run with the total of 214 world-wide modern populations and 485 ancient individuals grouped into 123 populations/individual samples. As expected the Admixture analyses showed that Polish Eneolithic individuals were carriers of three major components: West and North European HG (WHG; orange), Near East Neolithic (NEN; red), blue and green components of Asian origins (SA – South Asian - navy blue; NEA - North East Asian - light blue; SEA – South East Asian - light green and NSA –North/South American – dark green). In that respect, they were similar to earlier published Admixture analyses^11^ and revealed that individuals from Święte, Szczytna and Mirocin were carriers of larger NEN and SEA components than the representatives of Yamnaya. Polish CWC individuals also had traces of yet another component which is most prominent in Sub-Saharan and other modern African populations. According to earlier research the component was found to be more evident in Neolithic European populations than those with Steppe ancestry. In terms of differences between groups of individuals it seems that members of Group I carried larger proportion of SA and less of SEA component, while individuals in Groups III and IV had larger SEA and NEN components. This variation is mirrored by variation and component distribution patterns observed in previously published individuals form central European Neolithic (Figs. 4A, S23). Our findings point to variable local admixture patterns between earlier Neolithic populations from southern Poland and incoming Steppe nomads as well as structuring between CWC groups form different parts of present-day Poland.

### *f*_2_-, *f*_3_- and *f*_4_-statistics

We tested with outgroup *f*_3_-statistics, which ancient population/individuals shared more genetic drift with the ancient samples generated here individually. The different population size between the reference ancient population/individuals and the lack of significant results between closely related individuals and populations make it difficult to extract conclusions from these analyses.

Therefore, we grouped our ancient individuals into four groups (groups I-IV) based on the aforementioned geographical distribution of the sites and compared these groups between them and also with the ancient reference individuals and populations. Ancient reference individuals were merged into groups following the same criteria as in the ADMIXTURE analyses. Overall, although non-significant the results suggested a trend where the four groups share more genetic drift with Russia_Afanasievo than with Yamnaya and Groups I, II and III share more genetic drift with Poland_CW than with Russian_Afanasievo (Table S14 & Fig. S20**)**. This pattern was also mirrored by the *f*_2_-statistics.

To test for structuring in our sample set we performed multiple *f*_4_-statistics of a form *f*_4_(O, X; A, B) where O is the outgroup (Yoruba), X represent our test Eneolithic individuals divided into groups I-IV, and A & B represent populations tested for admixture. First, we tested for mutual relations between Polish Eneolithic groups from this study. While most of the results were statistically insignificant, we observed that group II was closer to group I & IV to the exclusion of group III (Z > |2|) confirming that Pełczyska would be a distinct population group. We observed that the significant *f*_4_ statistics were largely consistent with the grouping pattern on PCA (Table S16). When tested with other published European Neolithic populations we observed similar pattern in Late Eneolithic and Final Eneolithic populations, but not earlier ones, e.g. Globular Amphora. Groups I-IV are genetically closer to Russia_Afanasievo than to Yamnaya individuals (Z > |2.9|) and are also closer to Russia_Afanasievo than to each other (Z > |2.9|). Even when we compare two individuals from the same group, same chronology and same site with Russian Afanasievo with *f*_4_-statistics they share significant more genetic drift with Russian Afanasievo than with each other. These results indicate that there might be a bias in *f*_3_- and *f*_4_-statistics towards Russian Afanasievo. We do not observe this bias in the *f*_2_-statistics where individuals within groups are closer to each other than to Russian Afanasievo as expected. Due to low sample size of Afanasievo group (n = 5), which was further reduced by removing RISE507, likely the same as RISE508^11^ the observed affinity to the Russian Afanasievo should be interpreted with caution. To further investigate our observations and trace any bias that might result from the way reference data was generated, we perform the same *f*_4_-statistics calculation with division of Yamnaya dataset into whole-genome sequenced dataset (WGS) and enriched dataset (CP), we only trust the results of F4 stats using WGS Yamnaya data. We find that we still observe similar results to those obtained while using mixed reference dataset albeit with more similarities within WGS generated dataset (Table S16). Selected *f*_4_-statistics results are listed in Table S16.

### Kinship analyses (READ)

In order to check for kinship relations between individuals, we used Relationship Estimation from Ancient DNA (READ) between all pairs of individuals tested^40^ (Table S12). We identified two pairs of first-degree relatives in Święte (between the male pcw040 and boy pcv041 as well as the female pcw061 and the female pcw062), and a pair of second-degree relatives in Chłopice (the children in pcw211 and pcw212) (Tables S6). In further population based statistical analyses we exclude the individuals with lower coverage genomes from each related pair (i.e. pcw041, pcw062 and pcw212).

### Pairwise Nucleotide Diversity

We used pairwise nucleotide diversity to test for differentiation between pairs of individuals, groupings (pcw040/pcw070, pcw260/pcw280 and pcw361/pcw362 representative of Groups I, III and IV respectively) and compared the values with diversity estimated in between pairs of individuals representing various Neolithic cultural groups (Figs. 4, S2). On individual level, we found that pairwise nucleotide diversity is in line with results from READ. In other words, we pickup closer relations between three pairs of individuals which were also found to be related according to READ analyses (pcw04/pcw041, pcw061/pcw062 and pce211/pcw212. On group level, we discovered that nucleotide diversity is similar between the groups and is on par with diversity found in other Eneolithic cultures (Fig. 4B). We do not include Group II as it consists of individual from different sites and could result in artificially inflated diversity estimates.

### Functional SNPs

Finally, we selected the seven individuals with best genome coverage (pcw040, pcw061, pcw070, pcw211, pcw361 and pcw362), to test a number of functional SNPs, especially those associated with lactase persistence, eye and hair colour and a number of traits associated with Asian ancestry (Table S17). None of the individuals was a carrier of Asian ancestry alleles nor was any of the individuals lactose tolerant (Table S17). For those individuals who had enough coverage at pigmentation associated SNPs allowing for HirisPlex prediction, they were predicated to have been brown-eyed and dark (brown or black) haired (Table S18).

### Grouping individuals according to their provenance (Sr isotopes)

#### Local and nonlocal individuals

According to ^87^Sr/^86^Sr values some of individuals buried at sites in the Rzeszów Foothills are non-locals with signatures below 0.7103^41^ (Fig. 2B). While the dietary patterns of those individuals are consistent with CWC background, the archaeological and isotopic data indicate that their place of provenience (e.g. where they spent their childhood) could have been partly the Sokal Ridge or Ukraine. Using these data, and to test possible relations between the groupings of individuals we performed *F*_*ST*_ analyses using popstats^42^ between: A) the four groups as used for analyses in this paper (Groups I-IV); B) new grouping of four in which individuals from the Rzeszów Foothills were divided into local and non-local individuals, and where the latter were combined with individuals buried at sites in the Sokal Ridge (Group Ia, Group II-III and Group IVa); C) finally, we repeated the analyses by dividing the whole dataset into five groups (Group Ia, Group II-V), where group V consisted only of non-local individuals from the Rzeszów Foothills. We tested all possible groupings using SNP panels merged both with Human Origins and 1000 Genomes Project (Fig. S19). We find that inclusion of non-local individuals from Rzeszów Foothills in Group I makes the distance between that and the other groups lesser (Fig. S19A) than when the individuals are included in the alternative Group IVa (Fig. S19B). The general pattern of genetic distance remains the same and the results are reproducible between the two reference datasets used (Human Origins and 1000 Genome Project). When the dataset was divided into five groups (with individuals from Rzeszów Foothills divided into local and non-local group) we indeed observed that non-local individuals from Rzeszów Foothills are closest to individuals from the Sokal Ridge and both present very similar patterns in relation to other groups (Fig. S19C). However, since we detect no evidence of Group V constituting a uniform well-defined subgroup based on nucleotide diversity tests (Fig. S18) we find that grouping A, based on geography and archaeological context is most relevant for downstream genetic analyses and is thus used across different analyses.

## Discussion

All individuals studied here are associated to burials of the CWC and BBC complexes. Based on the geographic location and the strontium isotope analysis we identified four different territorial sub groups, and based on the genetics we identified at least two different groups within our sample set. The mtDNA and the Y-chromosome data provide a slightly different picture of the genetic variation in the region contrasting earlier studies of individuals from corresponding archaeological contexts from other regions of Central Europe. In contrast to observations by Juras *et al*.^12^ we did not find mitochondrial lineages specifically linked to Yamnaya pastoralists, instead most of mtDNA lineages found in our sample may be associated with European Neolithic farming groups as is the case for the Western Corded Ware sample in the earlier study^12^. Our results would indicate a stronger continuity with earlier Neolithic populations than previously observed. In other words, our study detected traces of an evident “incorporation” of local individuals into the migrating groups. However, the funerary rituals seem to have been affected in limited extent as the burials exhibit the typical CWC pattern in all cases examined. The Y chromosome haplogroup lineage R1b-M269 or R-L11 are characteristic of Yamnaya and Bell Beaker individuals^5,10^ and they were particularly widespread throughout Eurasia in the Bronze Age and thereafter^38^. Curiously, the haplogroup is uncommon among other published Corded Ware Complex individuals from Europe (Germany, Poland, Bohemia, Estonia, Lithuania)^6^ and is associated with the later Bell Beaker communities^5^. We see the inclusion of the Yamnaya genetic signals but again in a different manner than what has been shown in adjacent regions. These results indicate a higher level of CWC continuity with earlier Neolithic individuals than those previously studied. The result also shows that the CWC groups exhibit an influence of the Steppe world, i.e. in the individuals with specific Y chromosome. Later the influence of the BBC communities was stronger.

The PCA revealed that despite geographical proximity there is a distinct genetic separation between CWC and BBC individuals from southern Poland. The genetic variation of CWC individuals from southern Poland overlaps with the majority of previously published CWC individuals from Germany while the eight published CWC individuals from the Polish lowland^10,11^ more closely resemble BBC individuals (Fig. S21). This fact is not unexpected if we consider the CWC communities in Polish lowlands as representatives of north-western parts of the CWC world called as the Single-Grave culture (see supplementary information). The genetic variation of BBC individuals from south-eastern Poland overlaps with the broad variation of BBC individuals from Central Europe (Bohemia, Moravia, Germany, south-western Poland and Hungary) (Fig. S22**)** which corresponds well with archaeological data. The results are in line with Admixture analyses. To determine whether the structuring can be detected on even more regional level we divided our sample into regional subgroups to test their relations.

According to *f*_4_*-*statistics on individual level CWC individuals from south-eastern Poland are equally different to published CWC and Yamnaya individuals, while the three Pełczyska individuals tend to select German, Estonian, Lithuanian and Polish western CWC to the exclusion of Yamnaya (Table S16). This however is not the case for CWC individuals from the Kuyavia region published by Fernandes^11^. CWC (but not BBC) from south-eastern Poland tends to pick Yamnaya over CWC from Kuyavia region. Our results emphasize the different impacts the Yamnaya migration event had on different populations across Europe, i.e. the genetic legacy that the Yamnaya process left varies greatly between regions and cultures.

We obtained statistically significant values for group II being closer to group I & IV to the exclusion of group III confirming that BBC Pełczyska are a distinct population group. This suggests structuring not only within the Eneolithic in southern Poland but also between groups representing the same (Archaeological) cultural horizon. Compared to the Early and Middle Neolithic samples it seems that the CWC groups I, II and IV are equally distant from the Yamnaya pastoralists and from most of the earlier published Corded Ware groups from Estonia, Germany, Lithuania and central Poland (Table S16). The south-eastern Polish CWC individuals are significantly more closely related to Yamnaya than to CWC individuals from the Polish lowland supporting the differentiation between various CWC groups from Central Europe. This is in coincidence with archaeological finds that show differences between lowlands and uplands materials of CWC^23^. Bell Beaker individuals from Pełczyska mostly favour German, Polish (lowland) and Estonian CWC as well as German and Czech Bell Beaker populations over Steppe ancestors (Table S16). Interestingly, in contrast to CWC individuals from south-eastern Poland (Group I, II and IV), they share significantly closer affinity to Neolithic Iberian, Italian, Hungarian, Swedish, Polish TRB and Brześć Kujawski group populations (and nonsignificant but positive affinity to Polish Globular Amphora) than Yamnaya, pointing to possible continuity between this group and earlier populations. The genetic specificity of the population associated with this process shows similarity to the features of the BBC complex in Central Europe dated ca. 150-200 years later^5^.

Building on the idea that the CWC complex identity is founded on the type of burial rituals performed, the study of graves and the double burials in particular can be illuminating for interpretations of the local social structures of the CWC groups regarding possible kinship structures. Positive results were obtained from 3 double graves, all containing individuals of the same sex. Kinship was observed in the graves from Chłopice grave 11 (pcw211 and pcw212) and from Święte, grave 408 (pcw061 and pcw062). The former burial represented a second-degree kinship while the latter was a first degree one. The two young boys buried in the double grave from Łubcze (pcw361 and pcw362) were not closely related according our READ analysis. However, their similar Y chromosome haplogroup (Haplogroup R1b1a1a2a1a) leaves room for a possible shared ancestry on the male linage. In Chłopice both young females died and were buried at the same time but the cause of their death could not be established. Skeletons from Łubcze were too badly preserved to provide information on time or cause of death. Interestingly in Święte (grave 408), the younger female (pcw061) probably had died earlier than the older one (pcw062) as her remains probably were exhumed from another place and added to the grave with the older female. The niche construction made “revisits” and secondary depositions possible, however this practice was part of an uncommon funeral rite in which the kinship between the two females might have had relevance. Interestingly, these closely related females spent their childhood in different places as according to strontium isotopes analysis pcw061 was a non-local and pcw62 – a local individual in the area. The observations are particularly surprising and significant considering the CWC burial customs and also social organisation. They show that in some cases close relatives were buried together in spite of different time of death. Closely related individuals were also found in graves 40 A (pcw040) and 43 (pcw041) in Święte, although not placed in a double grave they had been placed in burials in close proximity of each other. Both individuals were non-locals. The first-degree kinship identified here most likely is the father and his son, as they both have the same radiocarbon date. The close proximity of the graves indicate that kinship was an important part of the society and may have been manifested even in the funeral customs of the Corded Ware Complex communities.

## Conclusion

Social processes in prehistory are hard to identify let alone interpret. Using ancient DNA and genomic analysis we can detect several levels of structuring and population dynamics within the CWC complex as well as between the CWC and BBC cultures present in Eneolithic south-eastern Poland. By evaluating the admixture between groups, the impact of earlier known demographic events like the Steppe expansions can be detected in our CWC individuals. Not surprisingly we also discover strong connections to the population in Central Germany and the CWC subpopulation living on the German lowlands. These ties are not new but they might indicate an interesting southern affiliation that has not been as clear in other previously studied samples from Poland. Furthermore, we detect less association between CWC groups from the Kuyavia region and southern Poland revealing fine scale routes of the spread of CWC traditions. The most unusual signal identified is the one between the CWC and the Afanasievo complex. This genetic incorporation from a Steppe population further east than the Yamnaya culture, is novel for these parts and suggests a CWC population structure and history more complex than previously thought. Although, our results should be treated with caution due to not just low number of samples, but the appearance of the same signal in individuals that predate steppe expansions, and are geographically more widespread. Our findings are in alignment with recent archaeological reviews suggesting lesser impact of Yamnaya event than estimated in earlier genomic studies (e.g.^16,17^). The CWC ancestry exhibits links both to common mtDNA linages of the initial Neolithic but also to those assimilating and replaced by the Yamnaya pastoralists. Moving further forward in time we detect genetic reminiscence of south-eastern CWC in BBC gene pool. This region was an important social area in the 3^rd^ Millennium BCE, a true prehistoric melting pot of human groups with different origin, which may have witnessed emergence of typical BBC genomics almost 200 years earlier than in other parts of Europe^5^.

By using strontium isotopes, we can detect locals and non-locals in our material, which can shed light on how different subgroups within the CWC interacted. Using these characterisations as a foundation allows for deeper look into kinship amongst the buried individuals. The identification of three different types of kinship amongst the individuals studied and especially in the context of an unusual burial customs expands our understanding of rituals and traditions underlying the social structures of the CWC complex.

## Methods

### Isotopic analyses

All carbon (δ^13^C_coll_) and nitrogen (δ^15^N_coll_) stable isotope analyses were performed at the Isotope Dating and Environment Research Laboratory at the Institute of Geological Sciences of the Polish Academy of Sciences in Warsaw^43^. Collagen was earlier extracted for AMS ^14^C dating in the Poznań Radiocarbon Laboratory. Stable isotope composition was determined using a Thermo Flash EA 1112HT elemental analyser connected to a Thermo Delta V Advantage isotope ratio mass spectrometer in a Continuous Flow system. The results of the carbon and nitrogen isotope analyses are shown in Fig. 2A and are listed in Table S5 (Supplementary Information). The stable isotope variation of the CWC individuals was compared to earlier published data on other CWC individuals from south-eastern Poland^29^. The Sr isotope analyses for 16 individuals were carried out in the Isotope Laboratory of the Adam Mickiewicz University at Poznań, Poland. The procedure included chemical separation of Sr and measurements of Sr isotope ratios. Strontium was analysed in dynamic collection mode on a Finnigan MAT 261 mass spectrometer. During this study, the NIST SRM 987 Sr standard yielded ^87^Sr/^86^Sr = 0.710236 ± 12 (2σ mean on 10 analyses). Total procedure blanks were less than 80 pg. The ^87^Sr/^86^Sr values were corrected to ^86^Sr/^88^Sr = 0.1194. The Sr results for samples were normalized to certified value of NIST SRM 987 = 0.710240 (Fig. 2B; Supplementary Information – Strontium Isotopes; Table S4).

### Ancient DNA extraction

53 archaeological bones were decontaminated prior to analysis by wiping them with a 1% sodium hypochlorite solution and DNA-free water. Furthermore, all surfaces were UV irradiated (6 J/cm^2^ at 254 nm). After removing 1 mm of the surface, a piece of the petrous bone was cut (50–150 mg) after which the bone was placed in an Eppendorf tube and left to dissolve in 1 ml extraction buffer (0.5 M EDTA and 10 mg/ml Proteinase K), at 55 °C in hybridization oven. The buffer was changed twice and all the aliquots were collected and combined. After one-week DNA was extracted following silica-based methods as in^44^ with modifications as in^45,46^ and eluted in 2 × 55 μl of EB buffer (Qiagen). One extraction was made from each sample and one extraction blank with water instead of bone powder was included per six to 10 extracts. Blanks were carried along the whole process until quantitative PCR (qPCR) and/or PCR and subsequent quantification.

### Library preparation and sequencing

DNA libraries were prepared using 20 μl of extract, with blunt-end ligation coupled with P5 and P7 adapters and indexes as described in^46,47^. From each extract one double-stranded library was built. Since aDNA is already fragmented, the shearing step was omitted from the protocol. Library blank controls (extraction blanks), were carried along during every step of library preparation. In order to determine the optimal number of PCR cycles for library amplification, qPCR was performed. Each reaction was prepared in a total volume of 25 μl, containing 1 μl of DNA library, 1X MaximaSYBRGreen mastermix, and 200 nM each of IS7 and IS8^47^ reactions were set up in duplicates. The amplification reactions had a total volume of 25 μl, with 3 μl DNA library, and the following in final concentrations: 1 X AmpliTaq Gold Buffer, 2.5 mM MgCl_2_, 250 μM of each dNTP, 2.5 U AmpliTaq Gold (Thermo Fisher Scientific, Waltham, MA), and 200 nM each of the IS4 primer and index primer^47^. PCR was done with the following conditions: an activation step at 94 °C for 10 min followed by 8–20 cycles of 94 °C for 30 s, 60 °C for 30 s, and 72 °C for 45 s, and a final elongation step of 72 °C for 10 min. For each library, six amplifications with the same indexing primer were pooled and purified with AMPure XP beads (Agencourt; Beckman Coulter, Brea, CA). The quality and quantity of libraries was checked using BioAnalyzer using the High Sensitivity Kit (Agilent Technologies, Cary, NC). None of the blanks showed any presence of DNA comparable to that of a sample and were therefore not further analysed. For initial screening, 10–20 libraries were pooled at equimolar concentrations for sequencing on an Illumina HiSeq X (2 × 150 bp, PE) at NGI Stockholm. After evaluation of factors such as clonality, proportion of human DNA, and genomic coverage samples were selected for resequencing, aiming to yield as high coverage as possible for each library. In this study we used single indexed libraries. While we acknowledge the issues that might be caused by index-hopping on Illumina HiSeq X flowcell, we strive to monitor for any issues by screening for presence of contamination

### Sequencing data processing

The sequencing reads were demultiplexed according to the sample associated index sequence. Paired-end reads were merged requiring an overlap of minimum 11 bp and minimum mapping quality of 30 and thereafter trimmed using MergeReadsFastq_cc.py^48^. Merged reads were mapped to the human reference genome (hs37d5) using BWA (v 0.7.8) with non-standard parameters (-l 16500 -n 0.01 -o 2)^49^. Files representing different sequencing runs were merged using samtools (v 1.8)^50^. Sequences with more than 10% mismatches, shorter than 35 bp and representing PCR duplicates were removed using FilterUniqSAMCons_cc.py^48^. Using R_y_ method which is based on the proportion of reads mapping to sex chromosomes genetic sex of the individuals were identified^37^. We re-mapped the published whole genome sequenced and SNP-captured ancient genome sequence data using the same parameters.

### Damage & Contamination

PMDtools^8^ was used in order to verify presence and quantify the proportion of 5’ cytosine deamination patterns which are characteristic of ancient DNA^30,32,51,52^. All individuals displayed damage patterns consistent with presence of ancient DNA (Fig. S17). To further evaluate data integrity, we estimated contamination levels by the analysing polymorphic site distribution in mitochondrial sequences and by investigating the likelihood of mitochondrial sequences mapping to more than one reference^33^ (Table S10). In addition to this, we estimated the degree of contamination in our samples using a likelihood-based method implemented in contamMix software^34^ that checks the matches of reads with 311 reference mitochondrial genome sequences from different worldwide populations.

### Mitochondrial DNA analysis

Sequencing reads with base quality of minimum 30 and aligning to mitochondrial DNA were filtered out using Samtools (v. 1.8)^50^. Consensus mitochondrial sequences were called using mpileup and vcfutils.pl (vcf2fq). Mitochondrial haplogroups were identified using HAPLOFIND^53^ and mthap v. 0.19b (https://dna.jameslick.com/mthap/) based on SNPs reported in rSRS and rCRS reference sequences respectively. All mutations and resulting haplogroup calls are listed in the supplement (Table S8).

### Y chromosome analysis

Sequencing reads with base quality of minimum 30 and aligning to Y chromosome were filtered out using Samtools (v. 1.8)^50^. Y chromosome sequences were called using mpileup. The resulting pileup file was then merged with haplogroup definitions from the PyloTree Y^54^. Y chromosome haplotype calls were reported using both PhyloTree Y nomenclature and the nomenclature of the International Society of Genetic Genealogy ISOGG database (https://www.isogg.org) v. 11.349 (Table S9). In order to avoid misidentification resulting from DNA damage whenever possible we base our haplogroup calls only on derived transversions.

### Reference datasets

We prepared 2 different datasets: Pseudo-haploid genotype calls of ancient individuals prepared by randomly choosing one read per position were merged with present day individuals from Human Origins data set^55,56^ and with 108 Yoruban individuals of the 1000 Genomes Project phase 3 data^39^. Human Origins dataset was used in PCA and ADMIXTURE analyses while 1000 Genomes Dataset was used in *F-statistics*. To prepare Human Origins Dataset, we discovered the genotypes of ancient individuals for a total of 594,924 autosomal SNPs genotyped for 2,730 modern-day individuals from 203 different populations and merged these ancient individuals as well as 485 ancient individuals collected from published data and processed with same parameters applied to our newly generated data (Table S13) with the present-day individuals. To prepare 1000 Genomes dataset, we discovered the pseudohaploid genotypes of ancient individuals for 1,938,919 transversion type SNPs with a minor allele frequency of 10% in Yoruba individuals in 1000 Genomes Project. To prepare this dataset, in brief, we first downloaded the VCF and BAM files of the African Yoruba individuals sequenced in phase 3 of the 1000 genomes project (ftp.1000genomes.ebi.ac.uk). Then we used vcftools and extracted all SNPs with a minor allele frequency of at least 10% in the Yoruba population. After which we excluded transition type SNPs to prevent potential bias due to postmortem damage in the analysis and lead us to retain a total of 1,938,919 SNPs^8,46^. For both datasets, the analyses were restricted to nucleotide positions with minimum mapping and base quality of 30. Indels and transition were excluded to avoid bias due to post-mortem damage.

### READ analysis

In order to identify possible kinship relations between individuals and to be able to exclude related individuals from downstream population analyses we performed READ (Relationship Estimation for Ancient DNA) analysis^40^. In order to guarantee the highest possible number of overlapping SNPs between individuals to ensure highest possible resolution of the analyses we merged our ancient individuals with a sub-sampled set of ten fully sequenced individuals from Estonian Genome Diversity Project (EGDP)^57^. The analyses were restricted to transversions only. After identification of three kin pairs (Table S12) from each pair we removed the individual with lower coverage to avoid bias in population analyses.

### Principal Component Analysis

The principal component analysis was performed using *smartpca* module in EIGENSOFT (v.6.0.1)^58^ with a selection of populations from the Human Origins dataset merged with ancient reference material^55,56^. We used *smartpca* with lsqproj:YES and shrinkmode:YES options and calculated the eigenvectors using only present-day populations. The resulting.evec files were visualised using Past 3.22^59^.

### ADMIXTURE analysis

In order to identify ancestral clusters, we performed unsupervised clustering using ADMIXTURE^60^. For this analysis, we used our data merged with the published ancient individuals as well as modern individuals from Human Origins dataset. Prior to analysis, we filtered the Human Origins dataset for linkage disequilibrium using PLINK^61^ with parameters “indep-pairwise 200 25 0.4”. To prevent bias due to difference in number of overlapping SNPs between ancient individuals we carried out ADMIXTURE analysis as described in^62,63^. First, we calculated ancestry components for present-day populations. Using the resulting ancestral allele frequencies, we estimated the cluster membership of each ancient individual. We run ADMIXTURE for K = 2 to K = 10 in 10 independent runs with different random seeds for modern individuals and used each of the resulting allele frequency file (P files) to determine clusters of the ancient individuals. We merged the resulting Q files for modern and ancient individuals and used Pong^64^ in greedy mode to align clusters between different runs and to identify common modes between various runs for each K run. The results were visualised using R (Figs. S22 and S23**)**.

### *f*_2_-, *f*_3_- and *f*_4_-statistics

In order to formally test for admixture, we calculated *f*_3_-statistics and *f*_4_*-*statistics using qp3Pop and *qpDstat* options of AdmixTools (v.5.0-20171024). The outgroup *f*_3_-statistics of the form (O; A, B) were used to check for shared genetic affinity between test populations, A and B^55^. We compared our ancient samples with 165 ancient populations/individuals (including the ones generated here) using Yoruban population from the 1000 Genomes data set as outgroup (O). The analyses were performed with 1938916 SNPs and 559 jackknife blocks (Table S14). The *f*_4_-statistics of the form *f*_4_(O, X; A, B) were used to check for tracing admixture between tested populations (Table S16). We computed *F*_4_ statistics setting the |*Z* | > = 2 as threshold. Since we compared population groups which are genetically very close to each other, we set the threshold as |*Z* | > = 2 and evaluated the genetic affinities between groups. Both *f*_3_-statistics and *f*_4_-statistics were calculated separately for all newly published individuals and for the same individuals grouped according to geographical clustering presented in Table 1. In all analyses, we exclude transition sites.

Finally, we used popstats (https://github.com/pontussk/popstats) to calculate nucleotide diversity between pairs of our individuals, FST between different groupings of our individuals (Fig. S18), *f*_2_-statistics between individuals, their groupings and different populations groups (Table S15) as well as conditional nucleotide diversity between pairs of CWC individuals representing three (out of four) different groups compared to a pairs of individuals representing selected contemporary population samples (Fig. 4B, Table S11)^42^. Standard errors were calculated using block jackknife over blocks of 500 SNPs (Tables S5, Figs. S17, S19, 4B).

## Data Availability

The sequence data was deposited in European Nucleotide Archive (ENA) under the following study accession number: PRJEB34091 (ERP116945).

## Supplementary information

Supplementary information.

Supplementary information2.
