# Supplementary material for: Corded Ware cultural complexity uncovered using genomic and isotopic analysis from south-eastern Poland
Source: Sci Rep. 2020 Apr 14;10:6885. doi: 10.1038/s41598-020-63138-w (PMC7165176; doi:10.1038/s41598-020-63138-w)
Supplement: Supplementary file 1 — Supplementary information. [file 41598_2020_63138_MOESM1_ESM.pdf]

Corded Ware cultural complexity uncovered using genomic and isotopic analysis from south-eastern Poland

Anna Linderholm1, *, Gülşah Merve Kılınç2, Anita Szczepanek3,4, Piotr Włodarczak3, Paweł Jarosz5, Zdzislaw Belka6, Jolanta Dopieralska7, Karolina Werens4, Jacek Górski8, Mirosław Mazurek9, Monika Hozer10, Małgorzata Rybicka11, Mikołaj Ostrowski12, Jolanta Bagińska13, Wiesław Koman14, Ricardo Rodríguez-Varela2, Jan Storå15, Anders Götherström2, Maja Krzewińska2, *

* Corresponding authors: Anna Linderholm (linderholm@tamu.edu)

Maja Krzewińska (maja.krzewinska@arklab.su.se)

Supplementary Information

1. The Corded Ware and the Bell Beaker societies in the Małopolska Uplands

Introduction

In the perspective of the analysis of archaeogenetic materials, the following remarks are initially the outline of prehistoric studies concerning the origin of the Małopolska (Lesser Poland) Final Eneolithic communities, and especially the contrast of autochthonic ideas and models assuming the important role of migrating mobile groups of pastoralists from the Eastern European steppe. Studies conducted on the Małopolska Final Eneolithic, from the Interwar Time corresponded with the most important European studies concerning this period. It was connected with the research procedure considering the tendencies in the approach to finds of Corded Ware culture (CWC) and Bell Beaker culture (BBC) developed in other countries. The consequence of this was that research efforts firstly were aimed to search for supraregional cultural and chronological determinants, which were primarily defined find types of mostly ceramic vessels and stone battle-axes. This was common in many countries (see e.g. [1] for a review)

Accordingly, the chronologically oldest find from Małopolska were considered to belong to the oldest Pan European CWC horizon (= horizon A, see [2-4]). This phase was followed by a phase of local CWC groups (see [5]) and, finally, in the last phase the appearance of BBC manifestations. The Final Eneolithic was treated primarily in a Central European perspective. However, still in the Interwar Period, further to the east, the shifted Polish borders resulted in research of finds from Małopolska together with materials from the forest-steppe zone of the North-Pontic area [6], which actually blurred the boundaries between the CWC and steppe cultures. The changed post-war situation resulted in the treatment of CWC and BBC as closed phenomena in a realm of a Central European world (eg., [7-9]). The "northern model" was usually used in the terminology labelling the period described here as the "late Neolithic", and less often as the "Final Neolithic", i.e. repeating the term often appearing in German literature. The term "Final Eneolithic" used here considers the intent of researchers from the southern regions of Central Europe (including the Czech and Moravian CWC). The term is better

synchronized with the terminology used for the Eastern European zone. Furthermore, the term is not only a determinant of the period, but also denotes a socio-cultural aspect (CWC and BBC), clearly distinguishable from the late Eneolithic system (in Małopolska: the Baden culture, the Globular Amphorae culture – GAC, and Złota type). The Małopolska finds from the Final Eneolithic are mainly graves. They were connected with CWC barrows and megalithic tombs (related to the TRB burial custom), as well as sometimes also to small, flat cemeteries.

The vast majority of sites are located on uplands covered with loess soils. This zone is clearly delimited from the north and west by lowland areas and from the south by the Carpathian chain. On the other hand, there is no clear border to the east: the loess-covered upland areas form the core of Volhynia and Podolia located in western Ukraine. The consequence of this is the presence of CWC cemeteries with features similar to those in the Małopolska province. However, most of them are discoveries from the beginning of 20th century and the knowledge about them is limited (inc. [6]). In the Małopolska region, the CWC graves are relatively numerous (currently: over 400), while the BBC graves are rare (in total: 32). The area studied here presents the most numerous and best-recognized group of Final Eneolithic finds in the eastern part of Central Europe. However, these data are clearly poorer than the richest agglomerations found in the western and southern parts (Bohemia, Moravia, Central Germany). The scientific value of the Małopolska finds has been enhanced by the increased number graves discovered during planned archaeological research, a relatively good preservation of skeletons - enabling the application of many types of specialist analyses, and the exceptionally rich grave equipment. The particular features of the funeral rituals included finds from various regions. In the case of Małopolska, however, their distinctive connection with the rituality of the communities living in the North-Pontic steppe/forest-steppe zone is noteworthy and of special significance for the present study.

Overview of the main stages of cultural development

Based on cemetery research, three stages of the Final Eneolithic are clearly distinguished. The oldest period (around 2800-2600/2550 BCE) is connected with the appearance of mounds of the older phase of the CWC. This period also includes the youngest GAC cemeteries and graves with transitional features of the Late/Final Eneolithic, called the "Złota type". The burial mounds of the older phase of the CWC are usually treated as an allochthonous phenomenon, and related to a migration of Final Eneolithic communities from the Eastern European zone (inc. [7, 10, 11]. The general idea of a central barrow grave - a chamber built most often with the use of wooden elements, and features of burial (such as the head orientation to the west, the crouched position on the back) finds analogies in the ideology of the steppe communities, including the Yamnaya culture. On the other hand, the details of the grave constructions, the lack of ochre application in the funeral ritual, equipment, technological and stylistic features of the finds were usually considered as local - Central European features (e.g., [7, 12]. Accordingly, it was assumed that all known CWC barrow graves from Małopolska did not represent the stage of migration, but probably were a consequence of that. At the same time, the possibility that steppe communities dispersed into Małopolska regions was indicated - starting from the turn of the fourth and third millennium BCE. This phenomenon, called the "CWC-X horizon" [10, 13, 14], would precede the rather static formalisation of the CWC

barrow ritual, i.e. the A horizon. Until recently, this was only a theoretical idea. Recently, this has been confirmed with the discovery of graves with skeletons coloured with ochre in burials at site 2 in Hubinek, dated to 3000-2900 BCE [15]: supplement; see also [16]. The barrow burials of the older phase of the CWC - both from Małopolska and from other regions of Europe - have not been the subject of archaeogenetic research so far. This is mainly due to the small number but also poor quality of bone materials that may be used to perform such analyses. The lack of archaeogenomic information hinders the interpretations of the demographic development and possible changes at the beginning of the Final Eneolithic period and the issue of the relations and developments between the CWC and the Yamnaya complexes.

Around 2600/2550 BCE new elements appeared in the funeral ritual of the Małopolska CWC societies, indicating contacts with the rites and practices of the Eastern European communities. The most important expression is the niche (catacomb) form of the grave construction. The idea – or analogies - of such a structure in the graves was already present in the earlier period in the Złota type cemeteries – ca. 2900-2600 BCE. In this phase specific types of constructions appeared with good analogies in the features of the Catacombnaya culture in the North-Pontic zone. Along with the new constructions, modifications of the burial arrangement, the orientation of the body and the burial equipment were made. Single graves dominate, although the number of double and multiple burials is noteworthy. In comparison with other regions of Central Europe, the Małopolska burials are equipped with particularly rich sets of gifts. Particularly specific is the nature of the equipment in some graves of adult men, which include ceramic vessels (the main type is a large beaker with a distinct neck), weaponry (stone battle-axes and arrowheads), sets of flint tools (mainly axes, knife inserts and strikers), bone, antler implements, stone implements (whetstones and grinding discs), copper tools and ornaments (including characteristic copper hair-rings), as well as “half-finished” items implements of flint. Thus, such burials emphasize the role of a man as a warrior, as well as a craftsman-specialist, whereas in the Małopolska materials the specialization in the field of flint making is especially emphasized. Unlike in other Central European CWC groups, at Małopolska there are numerous burials of men equipped with triangular arrowheads (up to 30 pieces). In other areas, the archery sets as an important element of the burial equipment of the deceased appears about 100-200 years later associated with the appearance and spread of the BBC ritual. Until recently, the researchers of the Małopolska Final Eneolithic have emphasized the differences between the older (with barrows) and the younger (with niche graves) CWC phases. In the latter, a significant interaction between the local cultural groups (such as TRB, Baden, GAC and Złota) has been noted, which is a contrast to the model of the older CWC phase (e.g., [7, 9, 12, 17, 18]. At the same time, the possibility of a local origin of the niche graves was acceptable [12], although in the 1960s there were voices emphasizing the importance of the eastern connections of these structures [19]. In the 1990s, as a result of the discovery at the site in Młodów-Zakącie [20] of an exceptional grave, renew attention was paid to the presence of the Middle Dnieper culture traits in the Małopolska graves [21]. Firstly, ceramic vessels such as the typical biconical beakers of "eastern" origin were identified. Then, also similarities between the equipment of the deceased in the Małopolska zone and those in the basin of the upper and middle Dnieper River was found. It concerned the presence of flint arrowheads in the male burials and rich tool equipment that highlighted the association of the flint technology and techniques of the

deceased. Based on the new observations, it was assumed that the migration of the Middle Dnieper culture communities to the Małopolska was an impulse that shaped and changed the nature of the funeral ritual and creating thus, created the differences seen in the burial rites of other regions of Central Europe.

The third stage of the Final Eneolithic is related to the appearance and impact of the Bell Beakers complex, which coincides with the disappearance of the CWC catacomb grave type (around 2400-2300 BCE). The BBC graves are located only in the western part of Małopolska [22]. The nature of the burial equipment and the way the burials are arranged correspond closely to the features found in other regions of Central Europe, firstly in the Moravian BBC. The process of cultural changes in the Final Neolithic has been associated to changes also in anthropological features of the deceased [23, 24]. Radiocarbon dates indicate a similar age of the BBC cemeteries and graves associated with the earliest stage of the early Bronze Age (the proto- and early phases of the Mierzanowice culture). However, the limited precision of the dating makes it impossible to detect a potential difference in more detail between the age of these two cultural phenomena. The burial equipment of the Mierzanowice culture, however, contains elements typical for the BBC (e.g., stone wrist guards). Vessels of the early phase of the Mierzanowice culture (mainly cups, jugs and pots) and their stylistic and technological elements also link the production to the bell beakers. Moreover, in this period associations and links to areas located in the eastern part of Małopolska and the Volhynia-Podolia Upland are clear, e.g. seen in the import of flints from the latter area. The funeral ritual of the early Mierzanowice culture contains elements of both culture complexes. Except for a general similarity to the CWC burial ritual, there are no specific features with links to the older Małopolska cemeteries: the details of the construction of the graves, the arrangement and orientation of the burials are different, and there are no specific elements of the Małopolska equipment (e.g. flint axes).

Economy and settlements

Traces of settlements and even camps connected with the CWC and BBC communities in the Małopolska area are rare. In the whole region, no traces of a multi-seasonal - permanent occupation have yet been found. This situation was an important argument for the interpretation of the mobile nature of the Final Eneolithic communities and the related economic profile based on breeding of large herds of animals (mainly cattle). These ideas were demonstrated in the settlement studies conducted in the spirit of processual archaeology (above all: [17, 25]). Additionally, a model was developed also for the gradual destabilization network of the (earlier) stable settlements in the fourth millennium BCE and the growing importance of livestock farming. In the spirit of these ideas, the emergence of the allochtonic barrow communities (CWC) in the first half of the third millennium BCE only accelerated the process of economic and social changes that had already begun, and they were determined at the same time by changes in environment. Attention was also paid to the importance of the climatic factors affecting the abandonment of the stable agricultural settlements located on the tops of loess uplands for the dynamic exploitation of the valley zones by smaller human groups [26]. A limited stabilisation of settlement, with the possibility of cultivating the land, is suggested for

the younger CWC period (around 2600/2500-2300 BCE), although the evidence confirming this process is still weak.

After approx. 2300 BCE in the Małopolska region, stable and sedentary agricultural settlements (the early stage of the Mierzanowice culture) appear again after a break of a few hundred years break. The mobile style of the settlement of the beaker societies is supported by the nature of the cemeteries left by them, forming small burial fields. Their features indicate that they were often familial or clan cemeteries. There are also cemeteries that spatially are connected with TRB megalithic tombs or earlier CWC barrows, which are a result of close but chronologically not immediately related episodes.

Context of aDNA investigations

The archaeogenetic analyses presented in this paper relate to burials associated with the second and third stage of Final Eneolithic in Małopolska, confirmed by the radiocarbon datings described below. The burials of CWC individuals in niche graves (16) do not correspond to the (hypothetical) oldest wave of the CWC phase with the assumed migration of pastoral communities from Eastern Europe (generally labelled the "A-horizon" or "Pan-European"). However, they are related to the second, separate phase (or "wave") of eastern influences, taking place around 2600-2500 BCE along the border zone between the forest and forest-steppe, and areas of Volhynia and Podolia towards Małopolska. The absolute dating of the burials analysed here correspond to the age of graves from other regions (including Central Germany or Polish lowland), from which burials have already been archaeogenomically studied. In this context, a certain genetic distinction of the Małopolska population from CWC individuals from other regions may be important. It is expressed by the repeatability of the Y-chromosomal R1b group in Małopolska materials, which has not been found in other regions of Central Europe but is present in the environment of Yamnaya and Catacombnaya steppe societies. The carried out comparative analyses indicated the closeness of the genetic pool of the Małopolska CWC and the Afanasievo culture. This challenging correlation, however correlates with archaeological conceptions assuming the eastern genesis of Catacombnaya culture, its significant influence on the formation of Middle Dnieper culture and then the expansion towards the west. Its consequence could be a modification of the gene pool, changed initially by the first migrations of the steppe communities towards the west. Both the published here and earlier results for BBC burials from Małopolska [27] indicate the presence of "steppe" haplogroups (including Y R1b). The "minor" difference in the genetic characteristics of BBC individuals from the slightly older Małopolska CWC, highlighted in the analyses is reflected in the prehistoric conceptions that assume the allochthonous character of populations with bell beakers. While assessing globally, it should be recognized that in the Małopolska zone, already in the late phase of the CWC (around 2600-2400 BCE) communities with genetic and cultural traits relatively close to the BBC model have appeared, which a little later (around 2400-2300 BCE) spread in the areas of Central and Northern Europe.

2. Materials

The successful DNA sequencing of a good quality was possible for 19 individuals (16 of CWC and 3 of BBC) from below described sites. All examined individuals come from different

geographical regions and they were grouped according to their provenience and cultural affiliation into groups I-IV (Fig. SX1; Table 1).

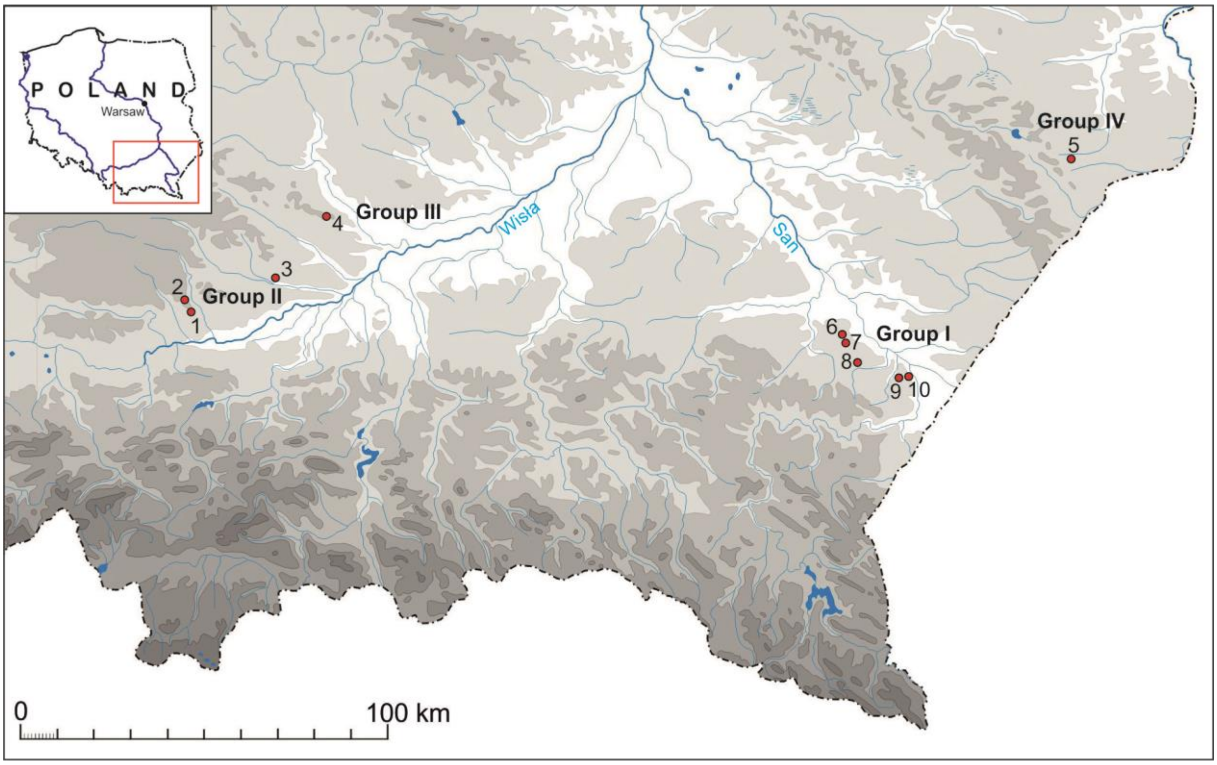

Figure SX1. The map of south-western Poland with marked location of archaeological sites and territorial groups: 1 – Kraków-Mistrzejowice, 2 – Bosutów, 3 – Proszowice, 4 – Pełczyska, 5 – Łubcze, 6 – Mirocin, 7 – Szczytna, 8 – Chłopice, 9 – Skołoszów, 10 – Święte. Insert shows location of the study area (red box) in Poland.

Group I: CWC graves in the Rzeszow Foothills (part of the Subcarpathian Region)

Święte, Jarosław district, site 11 (49°54’58N 22°51’39E)

Archaeological excavations were carried out in 2010 in connection with the construction of the A4 motorway by a consortium of Narnia-Archeo-Archgeo (research leaders: Aleksandra Łukaszewska and Łukasz Łukaszewski). An area of 37412 m^2 was examined. As a result, a burial ground of the Funnel Beaker culture was discovered, as well as 13 features (including 12 graves) of a cemetery of the Corded Ware culture [28]. In addition, a few traces of a settlement of the Bronze Age were registered (the Tarnobrzeska group of the Lusatian culture), relics of the intense settlement from the Early Period of Roman influence, and, finally fortifications from the First World War.

The site is located in the mesoregion of the Lower San valley, limiting the loess uplands of the Rzeszów Foothills from the east. It occupies the exposed western edge of the overflow terrace. The difference in height between the edge and the Holocene river valley is 12 m, and the altitude varies between 200 and 202 m above sea level.

The Corded Ware culture graves were discovered in two zones of the site. In the western part, the cemetery consisting of 10 features was located in the area previously occupied by the Funnel Beaker culture burial ground. However, at a distance of about 90 m to the east, at the margin of the excavated area, two graves were discovered that most likely belonged to another group of

burials, the majority of which were located outside the excavated area. A positive result of DNA analysis was obtained only for grave 876 **(pcw070)**, located in the western part of the site. It was a burial of a male connected with the younger phase of the Corded Ware culture, dated for about 2460-2340 BC.
The material is housed in the collections of the Rzeszów Archaeological Centre Foundation.

Grave 876
Grave 876 had a niche construction (Fig. SX2). The entrance pit was located to the south and led to the niche through a horizontal corridor. The niche had an oval shape and dimensions 200 x 150 cm. It had a homogeneous fill of dark brown colour. A well-preserved skeleton of an adult male aged 30-35 years lay on the bottom (120 cm), in the northern part of the niche. The skeleton laid on the back, with the head and lower limbs facing to the right side. Orientation was W-E with head to E. All equipment was located on bones of the pelvis and upper limbs (two beakers, a flint axe, a blade used as a knife and a bone chisel).

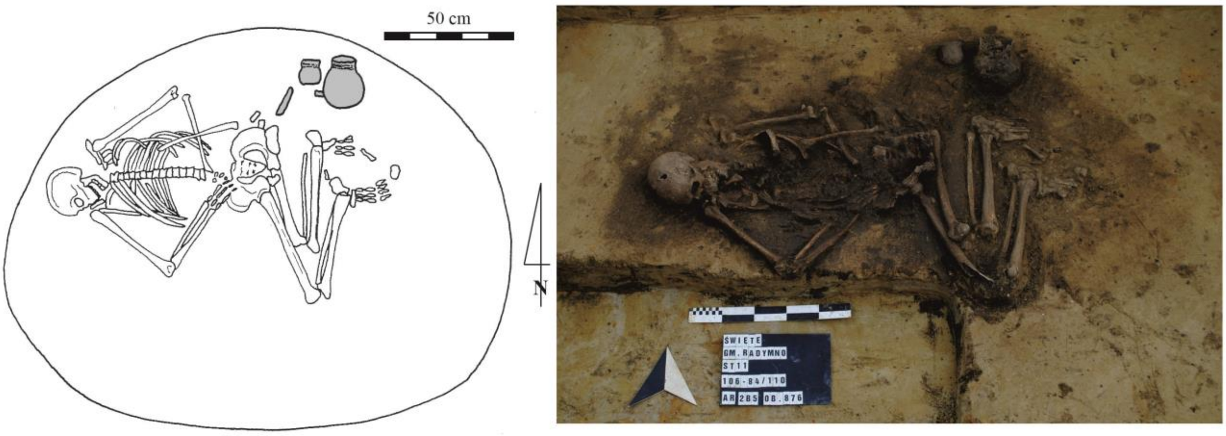

Fig. SX2. Święte, site 11, grave 876, plan of the burial.

Święte, Jarosław district, site 15 (49°54’49N 22°50’49E)
Rescue excavations preceding the construction of the A4 motorway were carried out in the years 2010-2011 by a consortium of Narnia-Arche-Archgeo (research leaders: Piotr Janczewski and Piotr Brożyna). A total area of 2.78 ha was excavated. In such a large area the number of finds dated to the prehistoric periods were small and limited to 9 features of the Corded Ware culture (including eight graves) and 10 features of the Trzciniec culture [29]. There are also documented tranches and other residues connected with the period of the First World War.
The site occupies the edge of the wide, Pleistocene terrace of the San River, falling down into the river valley by 12-15 m high sharp edge. This terrace is over 10 km wide, and its upper part is formed by a loess cover with a considerable thickness. On the whole area of the site a black earth humus level has been preserved *in situ*.
Most of the CWC graves were located in the eastern, only partially excavated part of the site. A single grave was discovered in the central part. A positive result of DNA analysis was obtained only for the double burial of females from the grave No 408 **(pcw061 and pcw062)**. This grave is connected with the younger phase of the Corded Ware culture and dated from

2460-2340 BC. The material is housed in the collections of the Rzeszów Archaeological Centre Foundation.

Grave 408

Grave 408 had a niche construction. The entrance pit was not possible to be documented. The niche had a circular shape and a diameter of 240 cm. Its fill was multi-layered and composed largely of yellow loess, indicating the collapse of the grave ceiling directly to the level of burial. At the bottom of the feature (about 120 cm from the ground level), in the Southern part, a relatively well-preserved skeleton of a female aged 30-40 years (burial B) was discovered lying in the left-sided position, head oriented to NW. A flint scraper was found on the epiphysis of the femur, and a small vessel near the bones of the vertebral column. In the northern part of the niche, there were scattered single bones of the second female, aged 20-30 (burial A) without anatomical order (Fig. SX3). Fragments of two vessels and a flint scraper were found next to it. In addition, at the level of the burial the bone awl and the metacarpal bone of the ovicaprid were found.

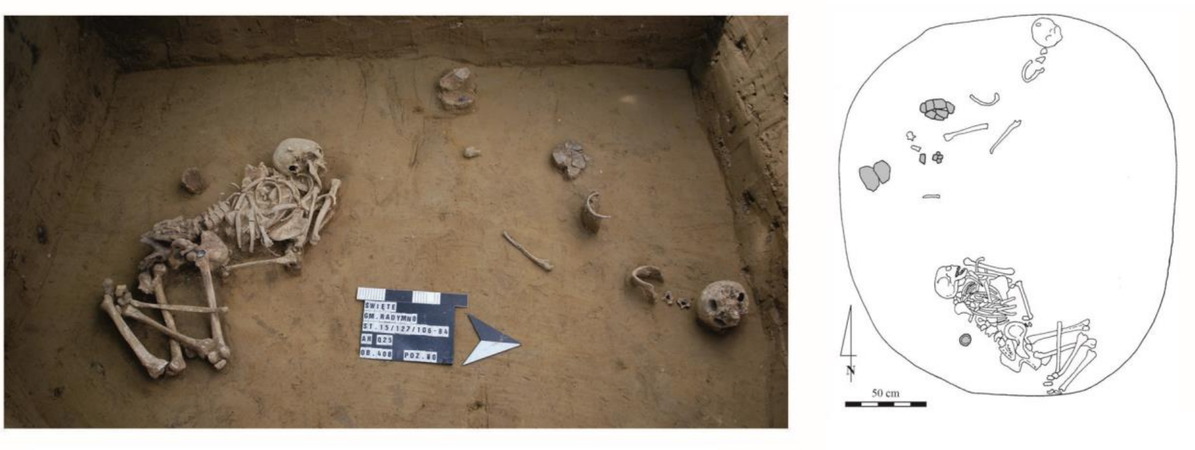

Fig. SX3. Święte, site 15, grave 408, plan of the burial.

Święte, Jarosław district, site 20 (49°54’47N 22°50’38E)

Rescue excavations preceding the construction of the A4 motorway were carried out in 2011 and 2014 by the consortium Narnia-Arche-Archgeo, and the excavations at site were directed by Agnieszka Zawadzka, Mateusz Królik and Paweł Greszata. An area of 5408 m^2 was examined, revealing the remains of settlements from the Neolithic period (the Funnel Beaker culture) and the early Bronze Age (the Mierzanowice culture), as well as remnants of activities from the First World War. Two graves of the Corded Ware culture that were located between the pits of the Mierzanowice culture were discovered in the prehistoric settlement zone [30].
The site is located within the Lower San valley – the mesoregion of the Sandomierz Basin. It occupies the edge of the Pleistocene terrace, elevated by about 12 meters above the bottom of the San River valley. It is located on a clearly separated spur, formed by erosive undercuts of the river meanders. The altitude of the terrain is 200-205 m above sea level.
Positive results of DNA analyses were obtained for both graves of the Corded Ware culture Nos. 40A and 43 **(pcw040 and pcw041)**. The features are connected with the younger phase of

the Corded Ware culture in the Rzeszów Foothills. Based on radiocarbon dating, their age was estimated to be around 2550-2450 BC. The material is housed in the collections of the Rzeszów Archaeological Centre Foundation.

Grave 40A

Grave 40A had a niche construction. The entrance pit with an elongated - oval shape was located to the south and led to the niche through a steep threshold. The niche had an oval shape and dimensions of 160 x 90 cm. At the bottom (70 cm) there was a poorly preserved burial of a child aged 7-9. It was laid in a contracted position, probably on the right side, head to NE (Fig. SX4). His equipment was a small beaker, laid to the east of pelvic bones.

Grave 43

Grave 43 had a niche construction. The entrance pit with an elongated, oval shape was located to the south side of the niche. Both elements were divided by the threshold of a few centimetres. The niche had an oval shape and dimensions of 220 x 190 cm. At its bottom (90 cm) a burial of a male aged 40-45 years was found, laid in a contracted position, on the right side, head to the West. To the East from skeleton scattered and incomplete remains of two other individuals were recovered: a woman aged 44-55 years and a child aged 7-8 (Fig. SX4). By the male's burial, an amphora, a beaker, two axes, a fire-flint and two flakes were laid. In the concentration with the bones of the other two individuals three cups, two axes and a whetstone were placed.

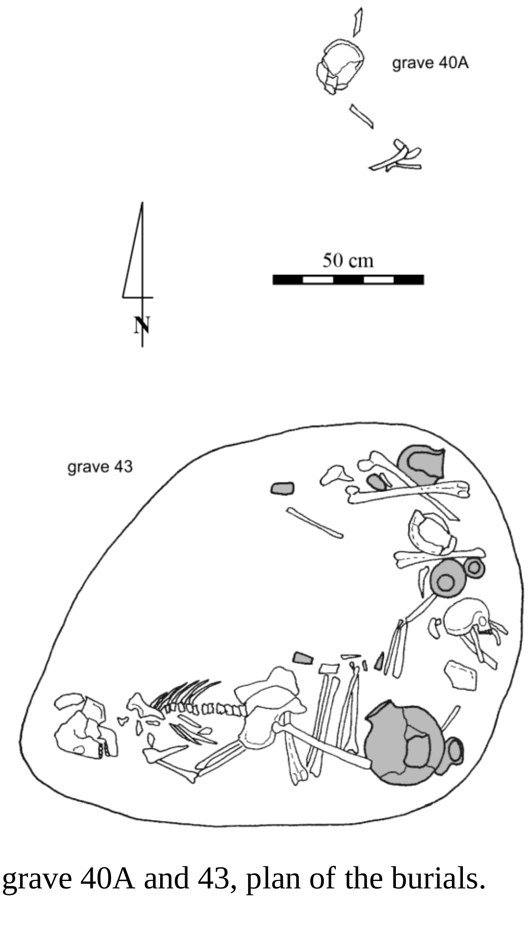

Fig. SX4. Święte, site 20, grave 40A and 43, plan of the burials.

Szczytna, Jarosław district, site 6 (50°0’43’’N; 22°35’39’’E)

The site was excavated in the seasons 2010-2011 [31] during the archaeological rescue excavations related to the construction of A4 Motorway along the Jarosław–Radymno section in south-eastern Poland. Fieldwork was directed by M. Hozer and A. Bajda-Wesołowska. It is a multicultural site occupied from the Neolithic to the Early Bronze period and then in the Modern Period. There were discovered 7 graves of the Corded Ware culture, five of them had a niche construction. DNA sequencing was possible only for one individual from grave No 84 **(pcw110)**. The material is housed in the collections of the Regional Museum in Rzeszów.

Grave 84

The outline of the niche grave was discovered at a depth of 40 cm (from the ground surface). At this level it had the shape of an elongated oval with dimensions 220 m (N-S) and 160 cm (E-W), and in the bottom part its size was 230 × 220 cm (Fig. SX5). The entrance pit led to a niche from the east side by a short corridor, 40-50 cm long and 60-70 cm in diameter, flowing smoothly towards the niche. The border between the entrance, the corridor and the niche were well-discernible, which indicates the use of some organic barrier. The niche had the shape of a rectangle with rounded corners and dimensions of about 166 × 142 cm. The remains of a child of the age of *Infant* I (5-6 years) were discovered at the bottom of the grave at a depth of 180 cm. The child was probably lying on the right side, and the equipment and the way the skeleton was situated indicate the burial of a boy. Behind the skull from the SW side bones of the rodent (*Rodentia*), snail shells of *Bradybaena fruticum,* and a fragment of the long bone of an animal of the size of cattle were found. In this part, a flint axe was also discovered. 46 beads from shells of molluscs belonging to the family of *Unionidae* family and 9 beads made from bones of an animal of cattle size were registered by the remains of the femur. In the NW and N part of the grave, i.e. behind the feet of the deceased, an amphora and a beaker were found. Three flint flakes were discovered between the vessels. A bony awl made of the metacarpal bone of the ovicaprids (*Capra/Ovis*) was also recorded. To the SE of the skull a miniature stone battle-axe probably intentionally broken was found.

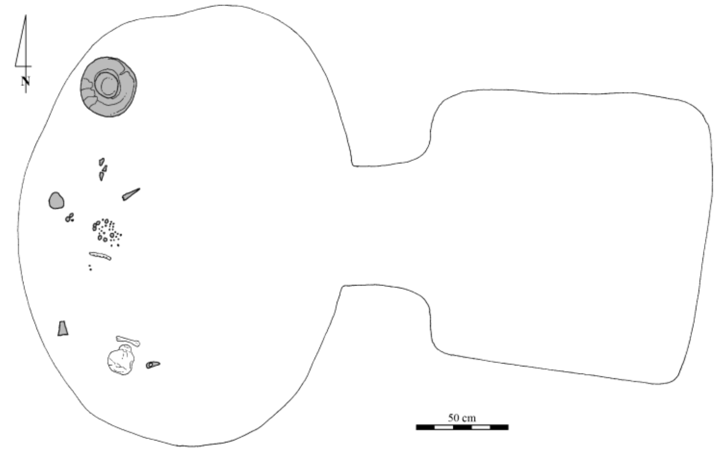

Fig. SX5. Szczytna, site 6, grave 84, plan of the burial.

Skołoszów, Jarosław district, site 7 (49°56’01’’N; 22°49’08’’E)

The site was excavated in the seasons 2010-11 during the archaeological rescue excavations related to the construction of A4 Motorway along the Jarosław–Radymno section in south-

eastern Poland. Fieldwork directed by M. Rybicka. It is a multicultural site occupied by The Funnel Beaker, Corded Ware and Mierzanowice cultures [32]. There were discovered 2 graves of the Corded Ware culture. DNA sequencing was possible only for one individual from feature No 256 **(pcw191)**. The material is housed in the collections of the Regional Museum in Rzeszów.

Grave 256

The burial was located within a concentration of archaeological features of a settlement and funeral character, it had dimensions: 470 x 420 cm and a depth of 160 cm.
Skeletons of two human individuals were found in the feature. The first one, individual I, was a child (7-8 years old) whose bones were dispersed in the northern part of the grave. The child's skeleton was not complete, and no (even) partial articulations were found. The second individual (II, a female, 20-30 years old) was partially in anatomical order – a skull, a vertebral column (up to Th10), a left rib cage, a left upper limb with the left-hand bones. The preserved elements showed that the individual rested on the left side. The bones of the right part of the rib cage and the right upper limb were disturbed, and the incomplete bones of the lower part of the skeleton were found scattered in the northern part of the grave and in the concentration in its western part (Fig. SX6). At the bottom of the grave between the bones belonging to the already mentioned female and child there were also skeletons of two goats [33]. The burial of the female was equipped with two vessels (an amphora and a beaker) and artefacts made of bone and antlers as well as a copper tube.

It was difficult to determine if the child's remains were originally buried in the northern part of the pit and, thus, the grave contained a double burial of a female and a child or whether the child's remains were chronologically younger and had been destroyed by the female niche grave of the Corded Ware. There was also a possibility that the remains of the child could have been "thrown" into the settlement feature of the Mierzanowice culture that was open for some time and in consequence its bones had been scattered in a pit. This was examined more closely by radiocarbon dating of the two individuals. The radiocarbon dates obtained for female’s rib gave the result 3915 ± 25BP (Poz-52608) which after the calibration for the probability range 95.4%, is 2474-2306 BC (the Corded Ware culture), while for the child's bone the result is 3830 ± 35 BP (Poz-55335), e.g. for a probability range of 95.4%, the result is 2457-2150 BC (and, thus, possibly the Mierzanowice culture).

Radiocarbon dating and the arrangement of human remains as well as a type of burial (probably a settlement pit) suggest that individual buried in Skołoszów (pcw191; see Supplementary: Materials) should be affiliated to the Early Bronze Age Mierzanowice culture (MC). DNA of this young female not significantly differ from DNA of other analysed CWC individuals, so it may be an example of genetic continuity between older Corded Ware culture and younger MC in its initial phase.

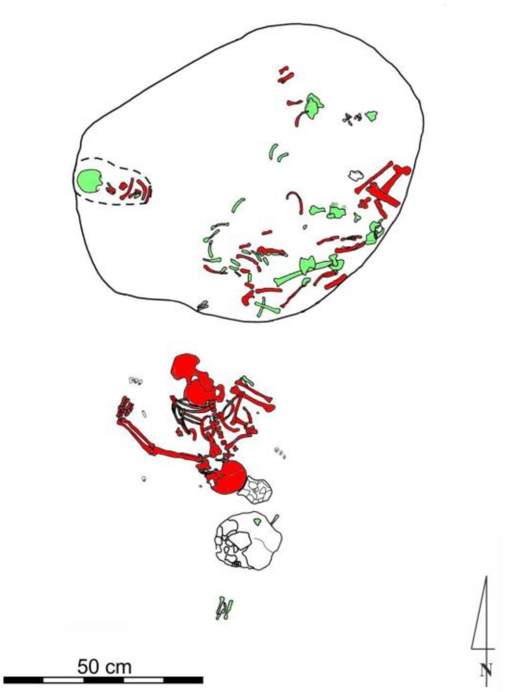

Fig. SX6. Skołoszów, site 7, grave 256, plan of the human burials: green bones – a child, red bones – a female.

Mirocin, Przeworsk district, site 27 (50º2’40’’N; 22º34’17’’E)
The site was excavated in the year 2011 during the archaeological rescue excavations related to the construction of A4 Motorway along the Jarosław–Radymno section in south-eastern Poland. Fieldwork directed by J. Okoński and M. Mazurek. The site is located at the culmination of the loess hump within the Rzeszów Foothills. It was occupied from the Neolithic to the Early Bronze period and then in the Modern Period [34, 35]. There were discovered 3 graves of the Corded Ware culture and one settlement feature of this culture. DNA sequencing was possible only for male individual from the double grave No 360 **(pcw160)**. The material is housed in the collections of the Rzeszów Archaeological Centre Foundation.

Grave 360
Grave 360 had a niche construction. At the level of discovery, it had a rectangular shape with rounded corners and dimensions 200x150 cm. The longer axis was oriented towards NE-SW. The flat bottom of the entrance pit was located at the depth of 70 cm from the level of discovery. Slightly oblique corridor, 70 cm long and 77 cm wide, led to a funerary niche which was located to SW from it. The dimensions of a niche of the grave were 275 x 185 cm. At its bottom (depth 115 cm from the level of discovery) there were two human burials. In the central part of the niche rested the skeleton of a male aged 50-60, laid on his back, and with his head and lower limbs facing the right side. Numerous skeletal elements were secondarily moved as a result of natural decomposition of the corpse and animal activities. The dead was oriented along the NW-SE axis with head to the SE, facing the entrance to the niche (NE). The second skeleton was located in the niche and its anatomical order was not preserved, occupying a space about 80 cm in diameter. This skeleton belonged to a female aged 45-55 years (Fig. SX7). Within the crypt,

rich equipment was discovered. About 65 cm to the west of the male chest there were 3 beakers, a stone battle-axe, two flint axes, retouched flakes, a flint knife and a wild boar's tusks. To the east of the skeleton at the distance of about 60 cm there were flint blades and flakes used as a knife. To the west of feet of the deceased there were eight flint arrowheads, a flint tool and three bone chisels. To the north of them there were vessels that surrounded the female bone concentration (Fig. 1, reconstruction). The miniature vessel was put into the amphora. Between these bones, copper applications and bone beads were found.

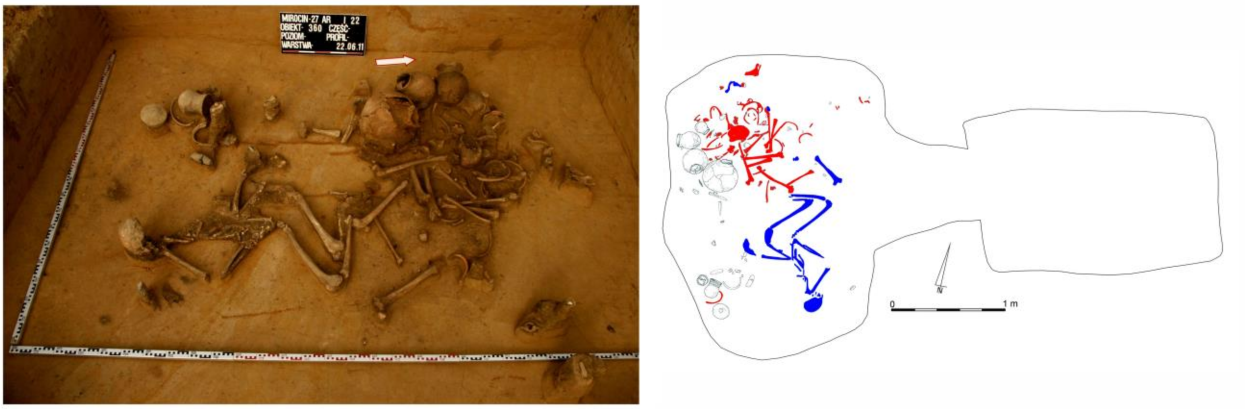

Fig. SX7. Mirocin, site 27, grave 360, plan of the burial.

Chłopice, Jarosław district, site 26 (49°57’26’’N; 22°39’37’’E)
The site was excavated in the year 2010 during the archaeological rescue excavations related to the construction of A4 Motorway. Fieldwork directed by J. Lepiejza. It is a multicultural site occupied from the Neolithic to Early Bronze period [36]. The site occupies the flattening of the loess foreland in the direction from east to west. There were discovered at least 3 sepulchral features of the Corded Ware culture. DNA sequencing was possible only for the double grave No 11 **(pcw211 and pcw212)**. The material is housed in the collections of the Rzeszów Archaeological Centre Foundation.

Grave 11
Grave 11 had a niche construction. The bottom part of the niche was oval in shape with dimensions of about 210 x 160 cm, and long axis was oriented according to the NW-SE line. At the bottom of the grave at a depth of about 85 cm two skeletons of girls aged 11-12 and 14-15 years resting on the left side (Fig. SX8) were found. Near the heads of both buried individuals was a beaker, and next to the feet of the older girl laid another beaker, and behind her, at the height of the pelvis, a bone awl and a flint tool from a flake. Between the heads of both individuals two pendants made from pierced animal teeth were registered, and below the feet of the younger child and slightly above the knees of the older one, 3 similar pendants of animal teeth were discovered.

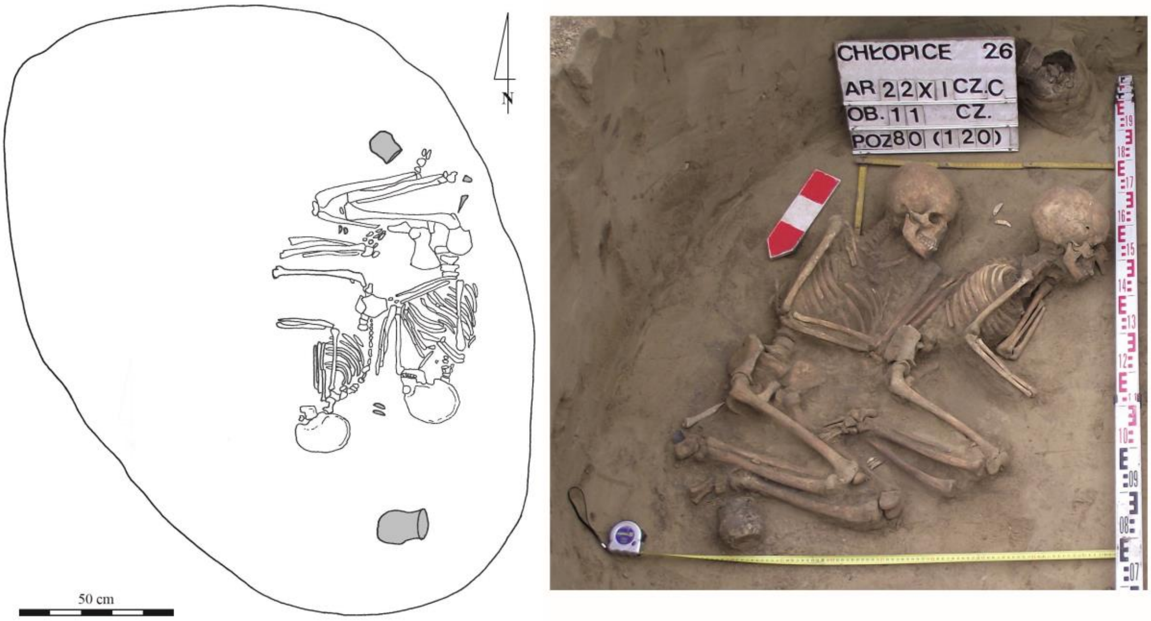

Fig. SX8. Chłopice, site 26, grave 11, plan of the burial.

Group II: CWC graves in the Małopolska Upland

Kraków-Mistrzejowice, Kraków district, site 85 (50º06’16’’N; 20º00’41’’E)

The site was excavated in the seasons 2008 and 2010-2011 [37, 38]. Fieldwork was directed by I. Mianowska and M. Ostrowski. It is a multicultural site revealing abundant finds of Neolithic occupations identified as Linear Pottery, Malice, Funnel Beaker, Baden, Corded Ware cultures and the Early Bronze Age - the Mierzanowice culture. Eight graves of the Corded Ware culture were discovered, and DNA sequencing was possible only for one individual from grave No 1311 (**pcw250**). The form of the graves and the depositions of all burials, correspond to the prevalent practices of the Kraków-Sandomierz Group [18]. The loess hill at Kraków-Mistrzejowice is the largest concentration of sepulchral features in the lower course of the Dłubnia River. These findings confirm intensive use of this area, both for habitation and for burial.

The material is housed in the collections of the Institute of Archaeology and Ethnology, Polish Academy of Sciences, Cracow.

Grave 1311

Grave 1311 had a niche construction (Fig. SX9). At the depth of ca 20 cm from the level of detection it measured 90 x 80 cm, its major axis-oriented N-S. The bottom of the entrance pit was found at a depth of ca 100 cm from the level of detection. To the west of the entrance was the niche of the grave. Near to the bottom of the niche its shape was oval, 230 x 190 cm (160 cm from the level of detection). The niche was not vaulted and had a height of ca 55 cm. At its bottom was a skeleton of a male, resting on its right side. The burial had been deposited along N-S axis, with the head to the south, the face towards the niche entrance (E). The upper limb bones had been moved by animals making their original arrangement unrecoverable. Grave goods were discovered next to the head and near the rib cage.

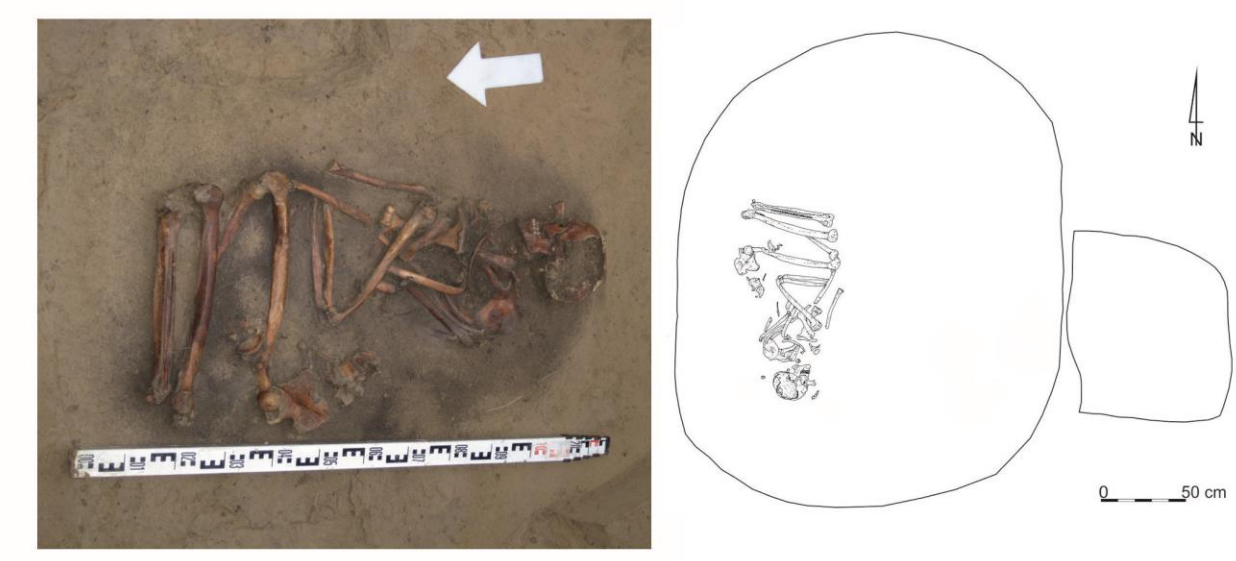

Fig. SX9. Kraków-Mistrzejowice, site 85, grave 1311, plan of the burial.

Proszowice, Proszowice district, site 1 (50º11’07” N; 20º17’57” E)

The excavations were carried out in 1963 by J. Prokopowicz [39]. Four destroyed graves of the Corded Ware culture were discovered at the site, found during the building of a hospital. DNA sequencing was possible for an individual from the grave 2 **(pcw420)**. The material is housed in the collections of the Archaeological Museum in Kraków.

Grave 2

Grave 2 had a niche construction and was destroyed in the upper part. The entrance pit was of the oval shape with the longer axis oriented along the east-west line with the dimensions of 200x130 cm with the bottom at a depth of 120 cm. A niche was also of oval shape, oriented along the north-south axis with dimensions of 300x260 cm, passed from the shaft from the west. Its bottom was at a depth of about 190 cm. Preserved bones allowed for the reconstruction of the body position of the deceased laying on the right side with the legs bent up and with the head to the south. Inside the niche equipment consisting of a beaker, a battle-axe, an amphora, an axe, and the blade and flake tools were found.

Bosutów, Kraków district (50º07’25” N; 19º59’35” E)

In 1956 three graves of the Corded Ware culture were accidentally discovered at the site [40]. DNA sequencing was possible for an individual from the grave 1 **(pcw430)**. The material is housed in the collections of the Archaeological Museum in Kraków.

Grave 1

Grave 1 was of a typical CWC type containing a male aged 40-50 was placed in the grave. He was placed in a contracted position with his head to the north-east. The grave was probably equipped with an amphora and a beaker located next to the skeleton.

Group III: BBC graves in the Małopolska Upland.

Pełczyska, Pińczów district, site 6 (50°21′22″N 20°34′17″E)

The multicultural site was excavated in 2001-2008 by Marcin Rudnicki from the Institute of Archaeology of the University of Warsaw. An area of about 0.7 ha was examined and documented, among others, graves from the Middle Neolith period (Funnel Beaker culture), Final Neolith (Corded Ware culture) and the Early (Bell Beaker culture and Mierzanowice culture) and Older Bronze Age (Trzciniec culture). The site is located on one of the vast chalk hills of the Wodzisław Hummock, covered with a thick layer of loess (the absolute height is 227 m above sea level). The graves of the Bell Beaker culture were located in the southern part of the excavated area, about 15 meters to the south of the hill zone occupied by the cemetery from the older periods of the Neolith [41]. DNA sequencing was possible for individuals buried in graves Nos. 12/2005 **(pcw260)**, 13/2005 **(pcw270)** and 25/2004 **(pcw280)**. The material is housed in the collections of the Institute of Archaeology and Ethnology, Polish Academy of Sciences, Cracow.

Grave 12/2005

The funeral pit in the horizontal section had a shape similar to a rectangle measuring 85 x 75 cm, oriented along the N-S axis. The fill was a layer of gray-brown, moderately compacted humus, mixed with small lumps of yellow loess. At the bottom (about 60 cm from the ground level), fragmentary preserved remains of a child aged 5-6 were discovered (a female - based on DNA analysis). The skull laid on the right side in the southern part of the grave, and in the northern part there were found fragments of long bones in a non-anatomical order were recovered (Fig. SX10). Between the remnants of the skeleton and the western edge of the pit, a bowl and a beaker were discovered.

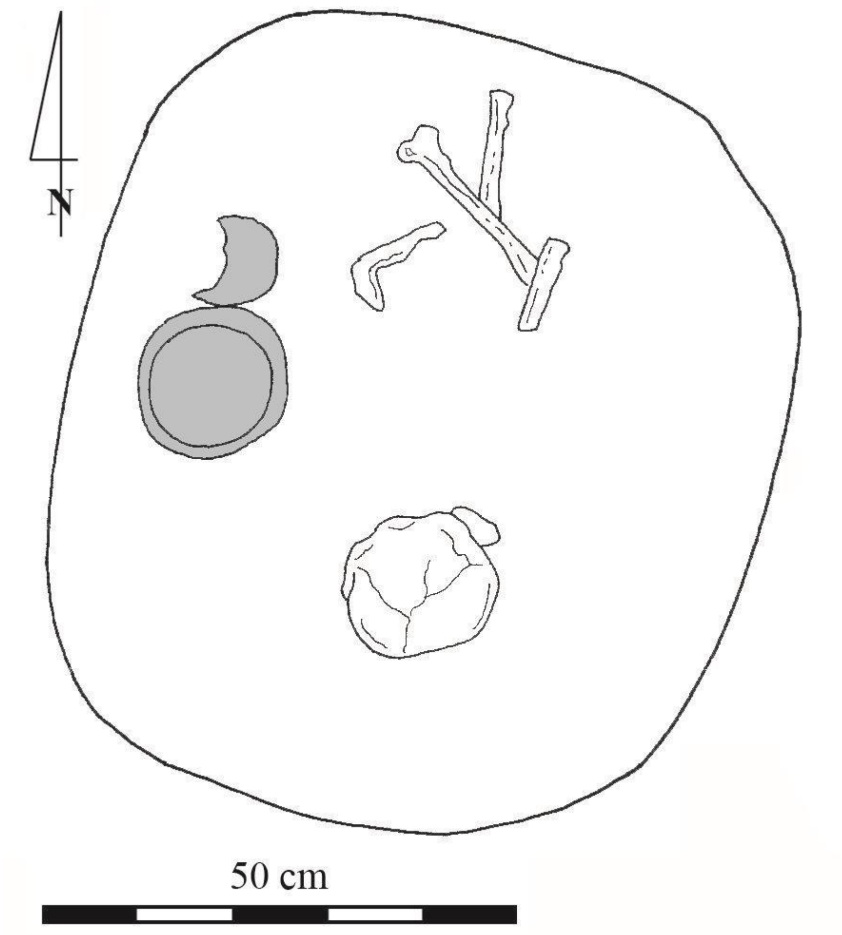

Fig. SX10. Pełczyska, site 6, grave 12-2005, plan of the burial.

Grave 13/2005

The grave pit in the horizontal section had an almost oval shape measuring 65 x 50 cm, with the long axis oriented along the N-S line. The grave fill was a layer of gray-brown humus mixed with lumps of yellow loess. At the bottom of the pit (about 40 cm from the ground level), in its southern part, a partially destroyed skull of the child was discovered at the age of about 2-3 years (female sex based on DNA analysis). In addition, several small fragments of bones, most likely belonging to the same individual, were found in the grave fill (Fig. SX11). Probably, the deceased was placed on the right side with head to S.

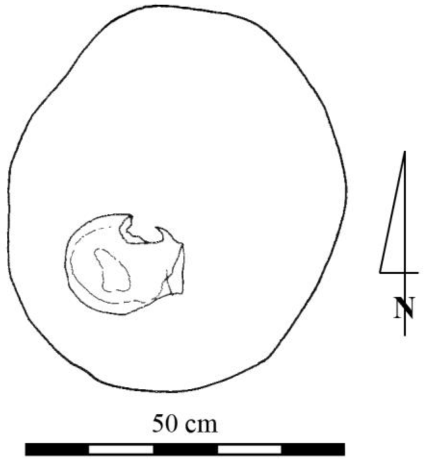

Fig. SX11. Pełczyska, site 6, grave 13-2005, plan of the burial.

Grave 25/2004

The burial pit had a shape similar to a rectangle measuring 55 x 45 cm and was oriented according to the N-S axis. The fill was a layer of brown, medium compacted humus, mixed with small lumps of yellow loess. At the bottom of the grave (about 40 cm from the ground) lay the remains of a child about 1-2 years old (female sex - based on DNA analysis). In the central part of the pit a fragmentary preserved skull was discovered (Fig. SX12). Directly by the east side of it stood a cup, and at the north-west edge of the pit a bowl was discovered. Small fragments of the postcranial skeleton were found during the exploration of the grave fill.

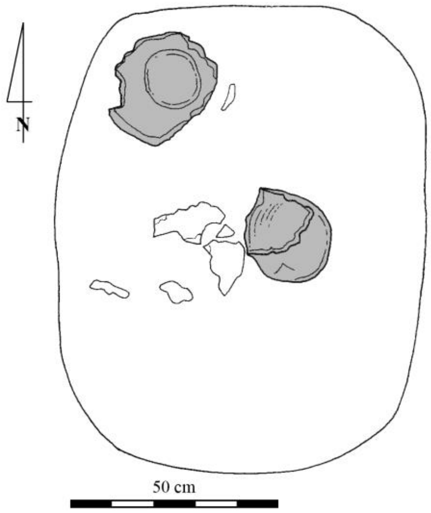

Fig. SX12. Pełczyska, site 6, grave 25-2004, plan of the burial.

Group IV: the Sokal Ridge (the western part of Volhynian Upland).

Łubcze, Tomaszów Lubelski district, site 2, barrow 2(50°27’22”N; 23°39’43”E)

The excavations of the barrow 2 were carried out in 1990, and it was located 30 m to the east of barrow No. 1 at the same site [21, 42]. The barrow was destroyed by ploughing, and the current height was only about 20 cm. Under the mound, two concentric furrows were found. They encircled the central grave. The subsequent graves (Nos. 2 and 3) were dug at the edge of

the mound. DNA sequencing was possible only for an individual from grave No 2 **(pcw350)**. The material is housed in the collections of the Regional Museum in Tomaszów Lubelski.

Grave 2

The grave had probably a niche construction. It was dug into a furrow at the south side of the burial mound. The niche was of the oval shape with the longer axis oriented along the NE-SW line, it was 200 × 150 cm large and 175 cm deep. A male aged 30-40 was buried and he was laid in a contracted position, on his right side, head to the West, and the face to the South (Fig. SX13). The burial equipment consisted of an amphora and two beakers located in the eastern part of the niche below the feet of the deceased, and a flint axe and a bone awl put at the height of his pelvis. From the bones of the burial, radiocarbon dating 4160 ± 50 BP (Ki-6298) and 3865 ± 35 BP (Poz-90898) were acquired.

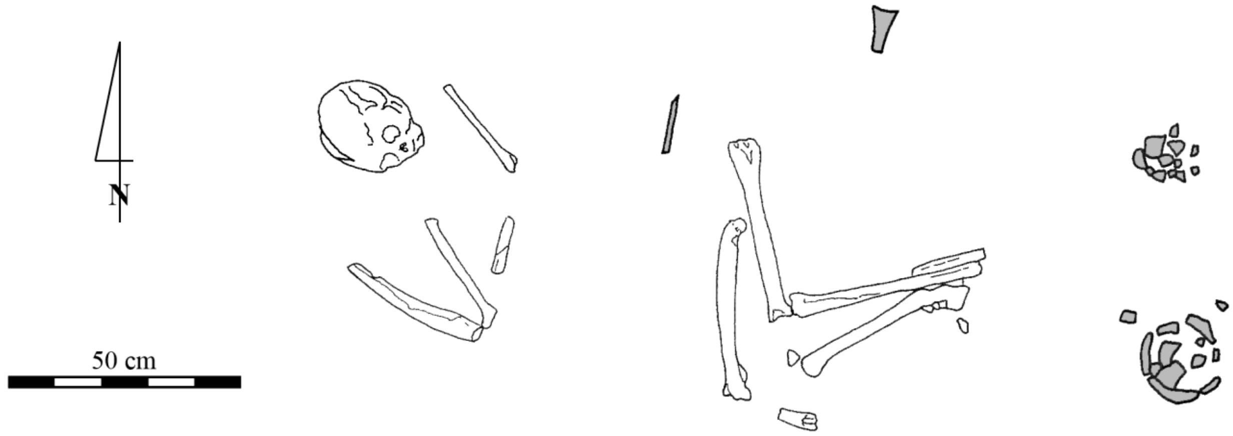

Fig. SX13. Łubcze, site 2, grave 2, plan of the burial.

Łubcze, Tomaszów Lubelski district, site 25, barrow 2 (50º27'22"N; 23º39'43"E)

Four barrows were registered at the site and all of them were excavated. They were erected on the local culmination of the Sokal Ridge. Barrow No. 2 was destroyed by ploughing, and was excavated in 1998 by W. Koman. Four graves were discovered under the remainder of the mound, including the central grave and the furrow surrounding the mound as well as the grave of the Mierzanowice culture and a settlement feature chronologically undetermined [42]. DNA sequencing was possible only for individuals buried in double grave No 3 **(pcw361 and 362)**. The material is housed in the collections of the Regional Museum in Tomaszów Lubelski.

Grave 3

The grave was located at the east side of the mound. The entrance pit had the longer axis oriented along the E-W line, and in the vertical section it was rectangular in shape and had the dimensions 140 × 120 cm and depth - 170 cm. A short corridor, going into an oval niche with a longer axis along the N-S line, passed from the shaft to the west. The niche had the dimensions 120 × 100 cm and height 60 cm, and its bottom was at a depth of 200 cm. By the southern wall of the niche there were burials of two children. Closer to the entrance to the niche there was a skeleton of a child about 3-4 years old, arranged in a contracted position probably on the left

side, and further in was the second child aged 4-5 years, arranged also in a contracted position probably on the right side (Fig. SX14). By the skulls in the southern part of the niche, three bone beads were found, and in the northern part, right at the entrance, two beakers and amphora were placed [21]. A radiocarbon dating of 3875 ± 35 BP (Poz-90899) was obtained for bones of the older child.

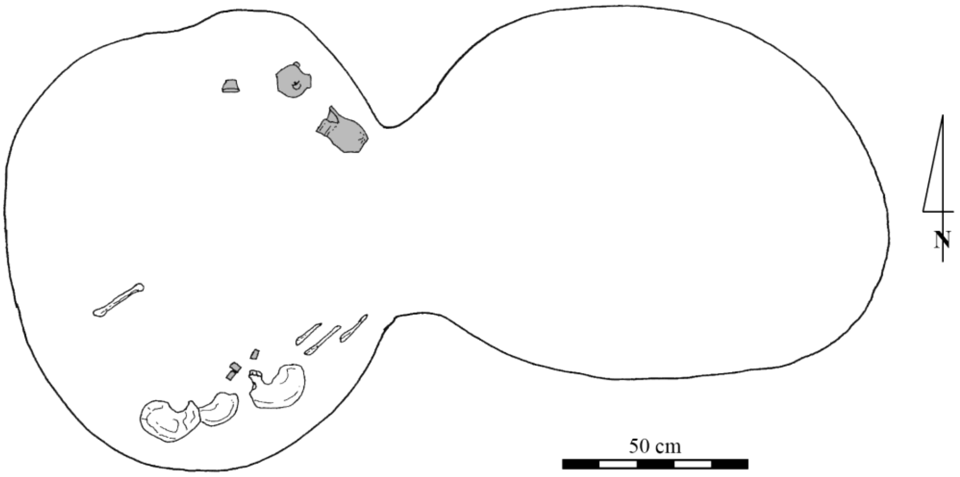

Fig. SX14. Łubcze, site 25, grave 3, plan of the burial.

3. Absolute chronology

Radiocarbon dates were made using the AMS technique for the bones of ten human burials: eight CWC, one BBC and one related to the initial phase of the Mierzanowice culture (Table SX1; Fig. SX15). The analyses were carried out at the Poznań Radiocarbon Laboratory. The obtained results are part of a longer series of dating made in this laboratory for Final Eneolithic graves from Małopolska (88 for CWC and 1 for BBC). Eight results for CWC were obtained for burials from niche (catacomb) graves combined with the younger phase of this culture. This series is characterized by compactness and indicates the age of all graves in the range of maximum 100-150 years: about 2500-2400/2350 BC. This is the period corresponding to the younger phase of the CWC in Central Europe, which includes the vast majority of graves. For this stage in south-eastern Poland, mainly niche graves are dated: dug into the burial mounds of the older CWC phase and TRB megalithic tombs, as well as creating flat burial grounds. Only a few of the CWC niche graves are slightly older and are from around 2600-2500 BC. Similar results of absolute dating were obtained at the Małopolska Upland for all macroregions: Małopolska Upland [26, 43], Lublin Upland [44], Sokal Ridge [21] and the Subcarpathian region [45]. The newly obtained AMS series of dating (mainly for human bones) are more precise than older results and revise previous chronometric examinations. On their basis, the absolute dating of the niche graves in Małopolska was corrected in relation to the previous findings assuming the early age of some of these finds - around 2800-2600 BC (e.g., [1, 46, 47]). In the light of these newer discoveries, the younger phase of the CWC is dated in Małopolska as in other regions of Central Europe and in Scandinavia [48]. The early stage of

the Final Eneolithic in south-eastern Poland is represented by CWC kurgans, as well as the flat cemeteries of the Złota culture. Their vast majority is dated to the range of around 2800-2600/2500 BC. In contrast to the cemeteries of the younger phase of the CWC, the number of burial graves available for chronometric and bioarchaeological tests is small. This makes it impossible to specify the chronology of the CWC phenomenon, and has not yet been able to obtain good quality aDNA research materials.

In the perspective of archaeogenetic analyses, the chronological relation of CWC niche graves to the kurgan cultural complexes from the northern Pontic zone, and above all to the Catacomb culture, is important. The absolute dating presented above unambiguously indicates that the graveyards of the younger phase of the CWC are younger than the graves of the Yamnaya culture. However, they correspond to the graves of the older phase of the Catacomb culture in the north-west area of the Black Sea region. Significant seems to be also the chronological relation of examined materials to the cemeteries of Afanasievo culture from western Siberia. The age of this phenomenon shows quite long-term duration: 3700/3400-2500 BC (e.g. Anthony 2007, 309). However, most of radiocarbon dating of this culture corresponds to the half of the 3rd millennium BC [49], 247, table 4), e.g. to the time of expansion of Catacombnaya trend and to the beginning of late CWC phase in Małopolska.

The classical and late phase of the Yamnaya culture is synchronous with the Małopolska kurgans of the early CWC, as well as with the graves of the Złota culture. The dating received for the BBC grave from Pełczyska indicates the interval of about 2350-2235 BC, which is younger than the late CWC phase in Małopolska. Similar results have recently been obtained for three BBC graves from Samborzec [27]. These results, obtained using the AMS technique, revised the earlier series of datings for this cemetery, which were clearly older and overlapped with the age of the late CWC phase [23]. These newer and more precise results of absolute dating for the BBC are the same as the ^{14}C dates obtained for funerary and settlement materials of the early phase of the Mierzanowice culture [50], and also slightly younger than the dating of the CWC in Małopolska. They refer to the generalized range around 2400-2200 BC. This is also confirmed by the age obtained for child's burial from the pit of the Mierzanowice culture in Skołoszów - identical to the dating of the BBC grave in Pełczyska.

Table SX1. The 14C measurements obtained for individuals published in this study.

**Site, No**	**Atlas ID**	**grave**	**Cultu -re**	**Age (years)**	**Sex (antr)**	**Sex (gen)**	**Lab No.**	**Age ^{14}C BP**	**Calendar age (BC) 68,2% (1σ)***
Święte, 20	pcw040	40A	CWC	7-9	M?	XY	Poz-90777	3950± 35	2479-2349
Święte, 20	pcw041	43/I	CWC	40-45	M	XY	Poz-90778	3950± 35	2479-2349
Święte, 15	pcw061	408a	CWC	20-30	F	XX	Poz-90780	3890± 35	2461-2351
Święte, 11	pcw070	876	CWC	40-50	M	XY	Poz-90875	3890± 35	2461-2351
Mirocin, 27	pcw160	360	CWC	50-60	M	XY	Poz-54043	3870±35	2459-2352

Skołoszów, 7	pcw191	256	CWC	7-8	?	XX	Poz-55335	3830± 35	2347-2235
Chłopice, 26	pcw212	11	CWC	11-12	F?	XX	Poz-90881	3985± 35	2492-2350
Pełczyska, 6	pcw260	12	BBC	5-6	?	XX	Poz-34734	3830±35	2347-2235
Łubcze, 2	pcw350	2	CWC	30-40	M	XY	Poz-90898	3865 ± 35	2458-2353
Łubcze, 25	pcw362	3/W	CWC	4-5	?	XY	Poz-90899	3875 ± 35	2459-2351

* Calibration according to OxCal program v4.3.2 of Ch. Bronk Ramsey from 2017, using INTCAL13 calibration curve [51].

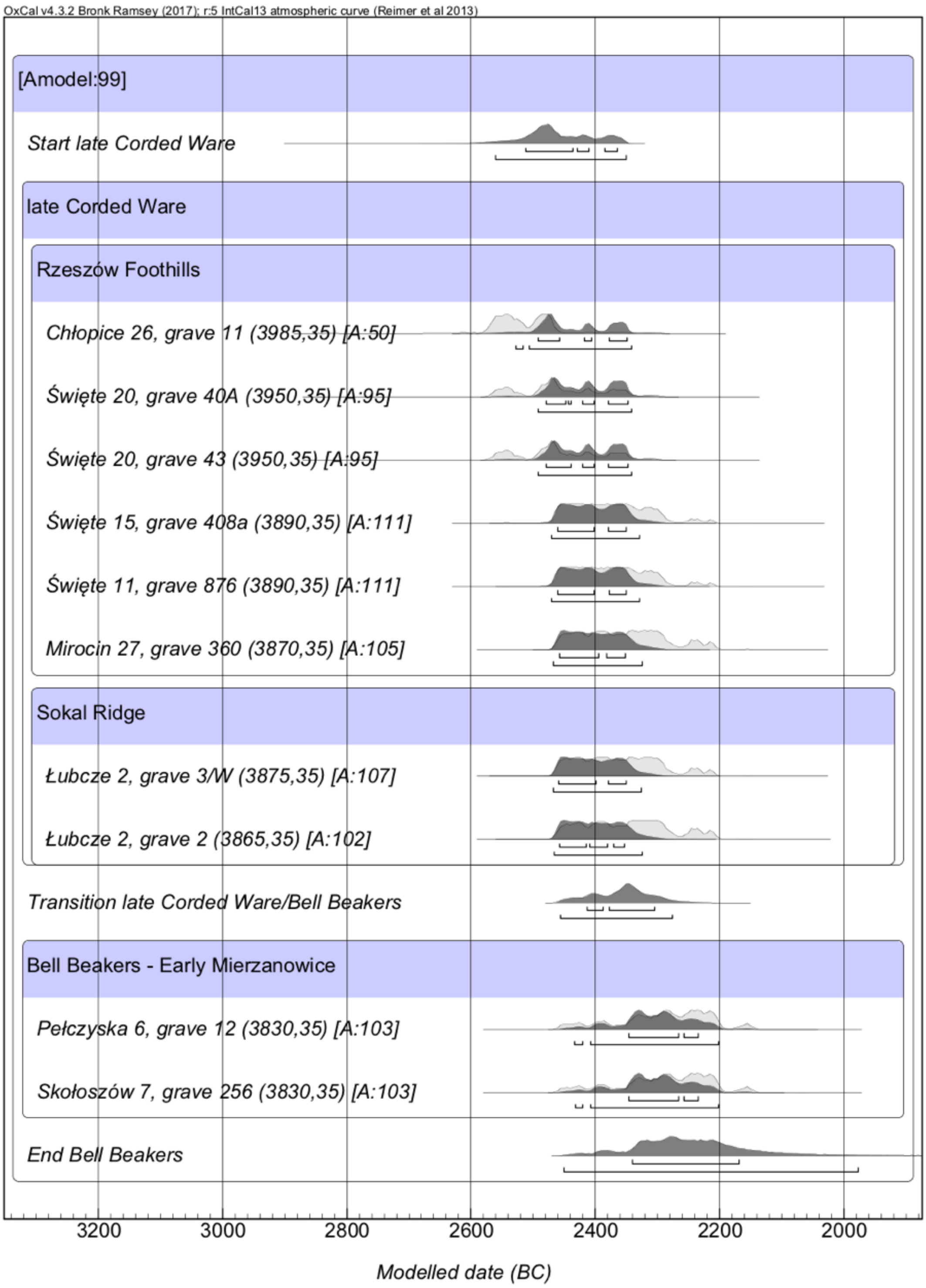

Figure SX15. A grave dating model assuming an older chronological position of CWC burials from the BBC burials and the early phase of the Mierzanowice culture.

4. Genetic verification of sex determination

According to latest summary of the burial customs of the Final Eneolithic communities [52] the typical feature of the Corded Ware funeral rite was to place the deceased placing in contracted positions mainly on the back or on the side with the lower limbs and the head turned to one side. Almost all female burials were positioned on the left-hand side and the majority of males

were laid on the right-hand side. Male burials were often equipped with items including battle-axes, arrowheads, sets of tools made of bone (chisels), and wild boar's tusks. At the cemeteries of Bell Beaker culture there is a clear change in these rules - males were interred on the left-hand side, and females on the right-hand side, that is, in the manner usual of the eastern province of the Bell Beaker cultural complex. Besides the changes in the placing of the deceased the Bell Beaker culture societies brought alteration in the type of deposited grave that reflected the new trends so many unique elements appeared. These were new types of vessels, copper and flint daggers, amber buttons with v-perforated holes, stone wrist guards and new types of copper and bone ornaments. These variations in funeral customs are interesting to compare and relate to archaeogenomic information in order to address earlier assumptions especially in cases of burials of younger individuals where morphological sex assessments are lacking (Table SX3).

Table SX3. Osteological, molecular and archaeological (side of the skeleton, grave goods) sex estimates obtained for individuals published in this study.

**Site, No**	**DNA lab ID**	**Grave**	**Culture**	**Age (years)**	**Sex (morph)**	**Sex (gen)**
Święte, 20	pcw040	40A	CWC	7-9	M?	XY
Święte, 20	pcw041	43/I	CWC	40-45	M	XY
Święte, 15	pcw061	408a	CWC	20-30	F	XX
Święte, 15	pcw062	408b	CWC	30-40	M	XX
Święte, 11	pcw070	876	CWC	40-50	M	XY
Szczytna, 6	pcw110	84	CWC	5-6	?	XY
Mirocin, 27	pcw160	360	CWC	50-60	M	XY
Skołoszów, 7	pcw191	256	MC	7-8	?	XX
Chłopice, 26	pcw212	11	CWC	11-12	F?	XX
Chłopice, 26	pcw211	11	CWC	14-15	?	XX
Mistrzejowice, 85	pcw250	1311	CWC	40-50	M	XY
Pełczyska, 6	pcw260	12	BBC	5-6	?	XX
Pełczyska, 6	pcw270	13	BBC	2-3	?	XX
Pełczyska, 6	pcw280	25	BBC	1-2	?	XX
Łubcze, 2	pcw350	2	CWC	30-40	M	XY
Łubcze, 25	pcw361	3/E	CWC	3-4	?	XY
Łubcze, 25	pcw362	3/W	CWC	4-5	?	XY
Proszowice, 1	pcw420	2	CWC	40-50	M	XY
Bosutów	pcw430	1	CWC	40-50	F	XY

According to presented data there is a visible compatibility in genetic and anthropological sex as well as archaeologically determined gender. Individuals of the Bell Beaker culture seem to respect the abovementioned customs linking with the position of dead. Unfortunately, all burials from Pełczyska are of young individuals and they are not well equipped. In the case of the Corded Ware culture there is a complete coincidence between the genetic sex and the archaeologically identified gender [52]. Two adult individuals show differences in the morphological and the genetic sex assessments. Such result for grave from Bosutów is

connected with a poor preservation of skeleton for which the complex sex determination according to standard methods [53] was not possible. The skeleton from the grave 408 in Święte demonstrated ambiguous criteria for sex assessments, and because of the massive and robust morphological structure it was anthropologically determined as a male.

5. Strontium isotopes

The results of the strontium isotope investigations are shown in Fig. 4B and listed in Table SX4. Tooth enamel of the CWC individuals from the Rzeszów Foothills gave a spectrum of $^{87}Sr/^{86}Sr$ ratios between 0.7098 and 0.7109 with one outlier at 0.7089. Six of the ten individuals were confirmed as non-locals with strontium signatures beyond 0.7104–0.7114 baseline established for the whole Rzeszów Foothills area [54, 55]. A high proportion of non-local individuals is a characteristic feature of the CWC communities in the area. Strontium isotope signatures below 0.7104 imply the presence of an unradiogenic bedrock component in the local environment, such as marine Neogene (Miocene) and/or Mesozoic carbonate rocks. The nearest areas that meet these conditions are located along the northern and eastern margins of the Carpathian Foredeep, i.e. north and east of the Rzeszów Foothills. Among these are Roztocze in Poland (a region where the Sokal Ridge is located) and areas in the basins of Dnieper and Pripyat rivers on the territory of the present-day Belarus and Ukraine. It is important to note that the presumed origin of non-local individuals from these regions corresponds well with the archaeological context [54]. At Mirocin, several allochthonous grave inventories, linked to the Middle Dnieper culture, were found [56].

The strontium isotope signatures of three male individuals from the Małopolska Upland (Tab. X4) are considered local to the region. The $^{87}Sr/^{86}Sr$ ratio of the child from the grave of the Bell Beaker culture at Pełczyska falls slightly below the local baseline range, being defined between 0.7095 and 0.7110. This appears to suggest its non-local origin. In the Pełczyska village, however, the local geology differs significantly from the bedrock of other archaeological sites in the Małopolska Upland. Here, the Maastrichtian (Upper Cretaceous) carbonates are locally exposed from under the cover of Pleistocene glacial deposits. It can be assumed that these carbonates likely show compositions that adhere to the marine Sr isotope curve characterizing the Late Cretaceous time, i.e. Sr signatures around 0.7078 [57]. It is, therefore, very likely that slightly lower $^{87}Sr/^{86}Sr$ signature of the child from the grave 12 may be due to the influence of unradiogenic strontium liberated by weathering from the Maastrichtian carbonates. In consequence, it seems to be the most probably that the child from Pełczyska was born locally.

Two individuals from the Sokal Ridge investigated during the present study gave relatively unradiogenic $^{87}Sr/^{86}Sr$ values of 0.7091 and 0.7094. These signatures fit well with Sr isotope data collected from other CWC sites located on the southern slope of the Sokal Ridge (Szczepanek, unpublished). They show a variation of $^{87}Sr/^{86}Sr$ ratios, from 0.7089 to 0.7105 (Fig. 4B). Unfortunately, there is no Sr isotope data so far from the bedrock of the Sokal Ridge. Its geological structure, however, is known in detail. This ridge, striking east-west, is made of up to 30 m thick cover of Pleistocene loess deposits overlying a substrate composed of Upper

Maastrichtian (Upper Cretaceous) marls and chalk. At the foot of the ridge, on its south side, there is a narrow, long valley filled with Holocene alluvial deposits, mostly sands and muds. From the Sr isotope curve of McArthur et al. [57] can be assumed that the Cretaceous carbonates should contribute unradiogenic strontium with $^{87}Sr/^{86}Sr$ values characteristic of the Maastrichtian, about 0.7078. In contrast, loess and alluvial deposits are certainly more radiogenic. Loess deposits that cover large areas in south-eastern Poland exhibit $^{87}Sr/^{86}Sr$ ratios above 0.72 [54]. Similarly, the siliciclastic composition of alluvial sediments points to radiogenic Sr isotope signatures, higher than about 0.715. Because of mixing processes in the hydrosphere, a local baseline should be positioned between the Sr composition of the Maastrichtian carbonates and those of the Quaternary deposits (loess, alluvial clastic sediments). The collected human enamel signatures (Fig. 4B) fit into such an interval. Therefore, it appears very likely that the variation of these signatures defines the local range in the Sokal Ridge. Compared to the Małopolska Upland, which includes similar lithological units in the bedrock, the local range in the Sokal Ridge is shifted towards more unradiogenic composition. This is presumably because the Maastrichtian carbonates are widely exposed south of the Sokal Ridge and they contain the main aquifer levels.

Table.SX4. Strontium values obtained for individuals published in this study.

**Site, No**	**Atlas ID**	**grave**	**Culture**	**Age (years)**	**Sex (gen)**	**Tooth**	**$^{87}Sr/^{86}Sr$ ±2 SD**
Rzeszów Foothills - CWC							
Święte, 20	pcw040	40A	CWC	7-9 *	XY	UM1	0.709889±10
Święte, 20	pcw041	43/I	CWC	40-45 *	XY	UM1	0.709818±15
Święte, 15	pcw061	408a	CWC	20-30 *	XX	LM1	0.710060±10
Święte, 15	pcw062	408b	CWC	30-40	XX	UP1	0.710706±12
Święte, 11	pcw070	876	CWC	40-50 *	XY	LP1	0.709804±08
Szczytna, 6	pcw110	84	CWC	5-6	XY	LM1	0.710884±10
Mirocin, 27	pcw160	360	CWC	50-60 *	XY	LM1	0.708881±14
Skołoszów, 7	pcw191	256	CWC	7-8 *	XX	UM1	0.709854±10
Chłopice, 26	pcw212	11	CWC	11-12	XX	LM1	0.710495±10
Chłopice, 26	pcw211	11	CWC	14-15	XX	LP1	0.710853±10
Małopolska Upland - CWC							
Mistrzejowice, 85	pcw250	1311	CWC	40-50	XY	LM1	0.709933±16
Proszowice, 1	pcw420	2	CWC	40-50	XY	LM1	0.710614±09
Bosutów	pcw430	1	CWC	40-50	XY	LM1	0.710795±10
Małopolska Upland - BBC							
Pełczyska, 6	pcw260	12	BBC	5-6	XX	LM1	0.709384±16
Sokal Ridge - CWC							
Łubcze, 2	pcw350	2	CWC	30-40	XY	UM1	0.709128±14
Łubcze, 25	pcw362	3/W	CWC	4-5	XY	UM1	0.709382±10

* – non-local individuals

6. Stable isotopes – diet

The results of the carbon and nitrogen isotope analyses are shown in Fig. 4 and are listed in Table SX5. Individuals with DNA analysed were presented on the background of other Corded Ware culture individuals from south-eastern Poland (acc. to [54, 58], and some unpublished data acquired during investigations within projects No. NCN 2015/19/B/HS3/02149 and 2016/20/S/HS3/00307)

Table SX5. Results of stable isotope analyses of human bones.

**Site, No**	**Atlas ID**	**grave**	**Culture**	**Age (years)**	**Sex (antr)**	**Sex (gen)**	**$\delta^{13}C$**	**$\delta^{15}N$**
Święte, 20	pcw040	40A	CWC	7-9	M?	XY	-20	11.9
Święte, 20	pcw041	43/I	CWC	40-45	M	XY	-20.1	11.5
Święte, 15	pcw061	408a	CWC	20-30	F	XX	-19.9	11.2
Święte, 11	pcw070	876	CWC	40-50	M	XY	-20.2	11.9
Mirocin,	pcw160	360	CWC	50-60	M	XY	-20.4	9.5
Chłopice,	pcw212	11	CWC	11-12	F?	XX	-20	11
Łubcze, 2	pcw350	2	BBC	30-40	M	XY	-19.9*	10.9*
Łubcze,	pcw362	3/W	BBC	4-5	?	XY	-20*	10.5*

*data unpublished earlier

The human stable isotope values of individuals with sequenced DNA except one outlier vary within a narrow range: $\delta^{13}C$ between –20.2 and –19.9‰ and $\delta^{15}N$ from 10.5 to 11.9‰. This one individual (pcw160) has the lowest signatures of carbon and nitrogen isotopes. All domestic animals from the same region demonstrate depleted levels of both $\delta^{13}C$ and $\delta^{15}N$ values. Acquired data are usual for humans living in a moderate climate terrestrial diet based on C3 plants and animal protein, with possible riverine resources consumptions [36, 45]. Other CWC representatives from south-eastern Poland revealed more variations (Fig. 4) and detailed analysis of individuals buried in graves at the Rzeszów Foothills showed difference in $\delta^{15}N$ values between males and females, with the male diet possibly having a more significant riverine component than the female diet [58]. Significantly differ are only young individuals with $\delta^{15}N$ values 13.5‰ and more that is an example of breastfeeding effect.

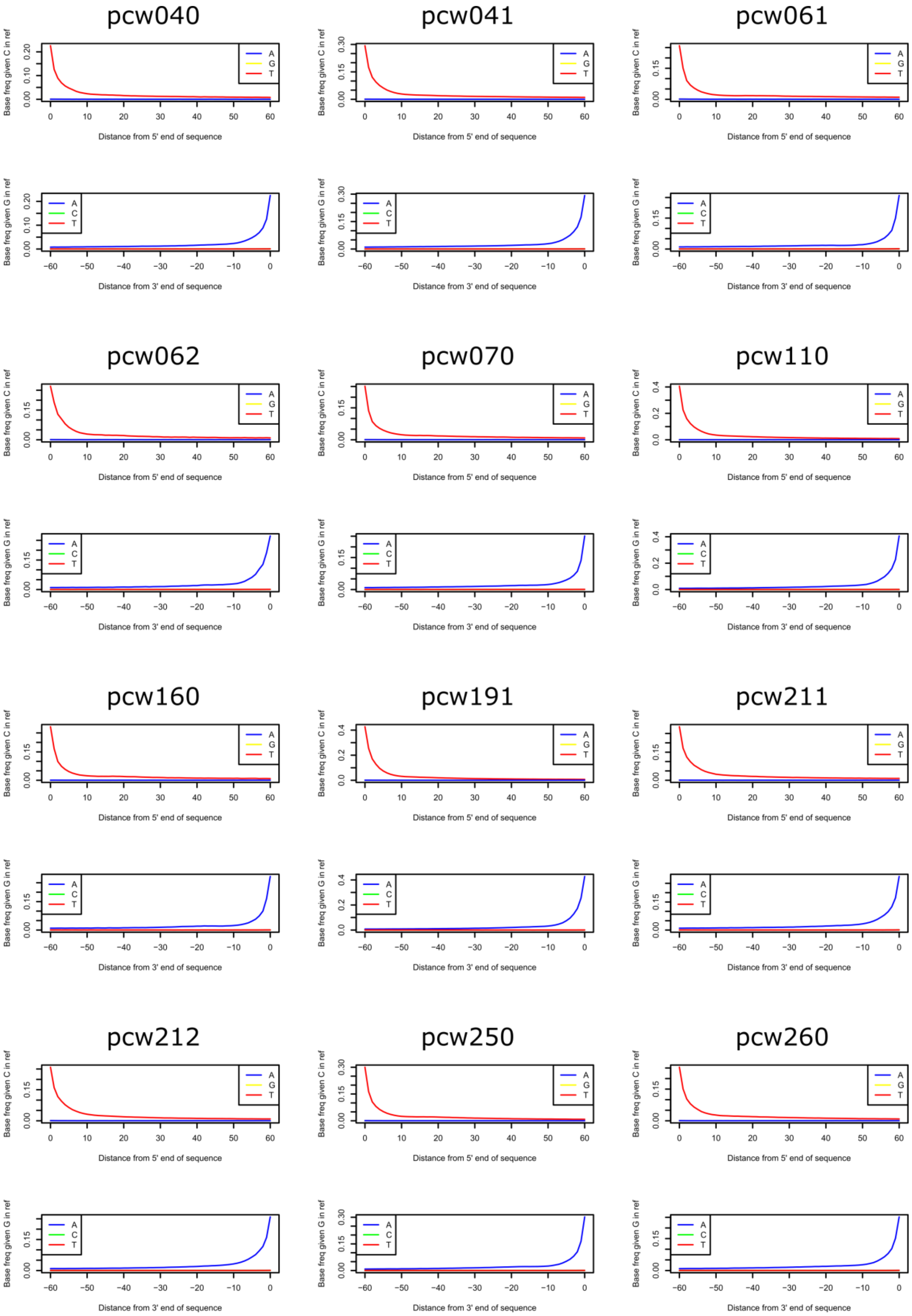

Figure SX16. Damage pattern plots for all individuals.

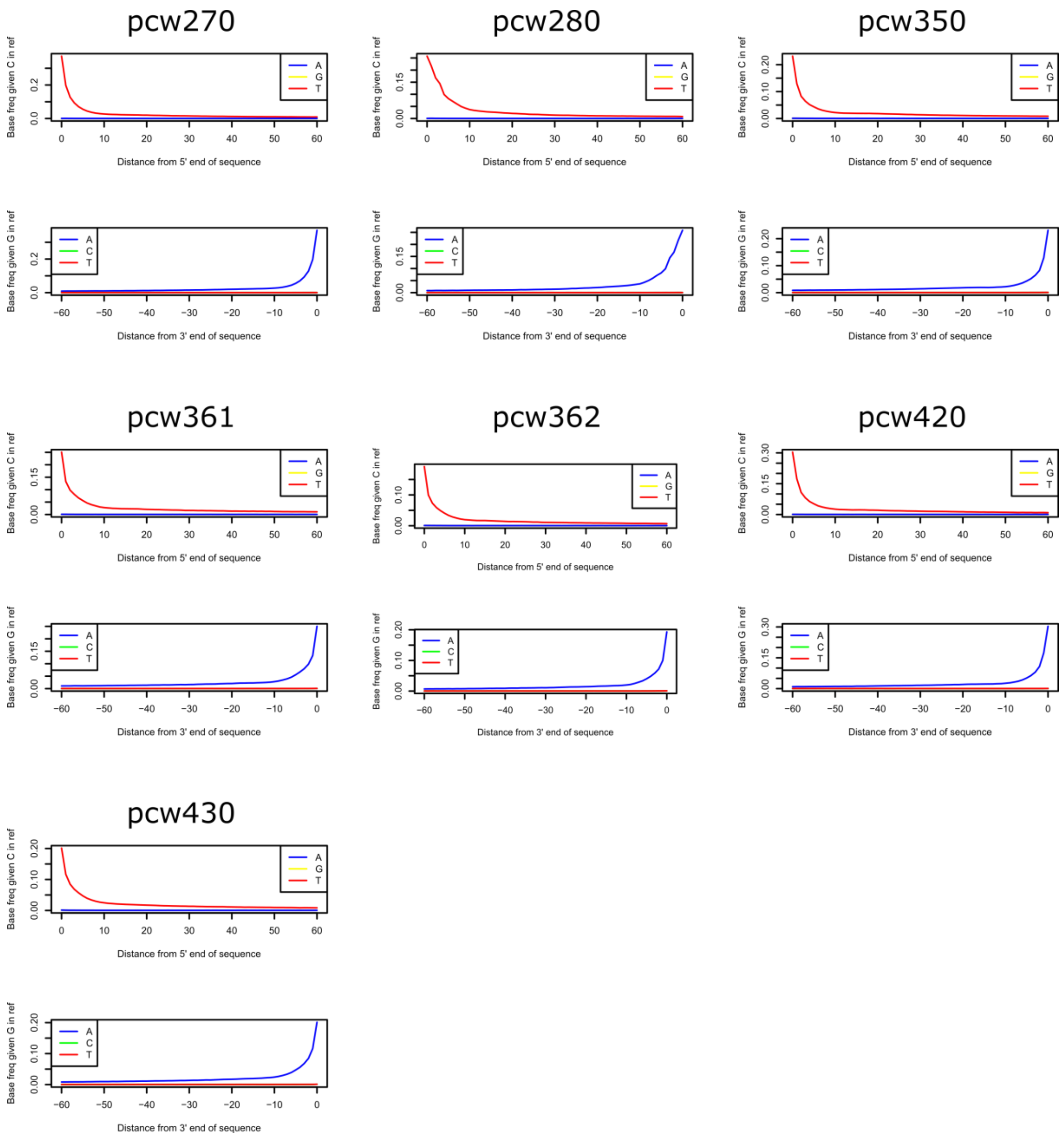

Figure SX16. Continued.

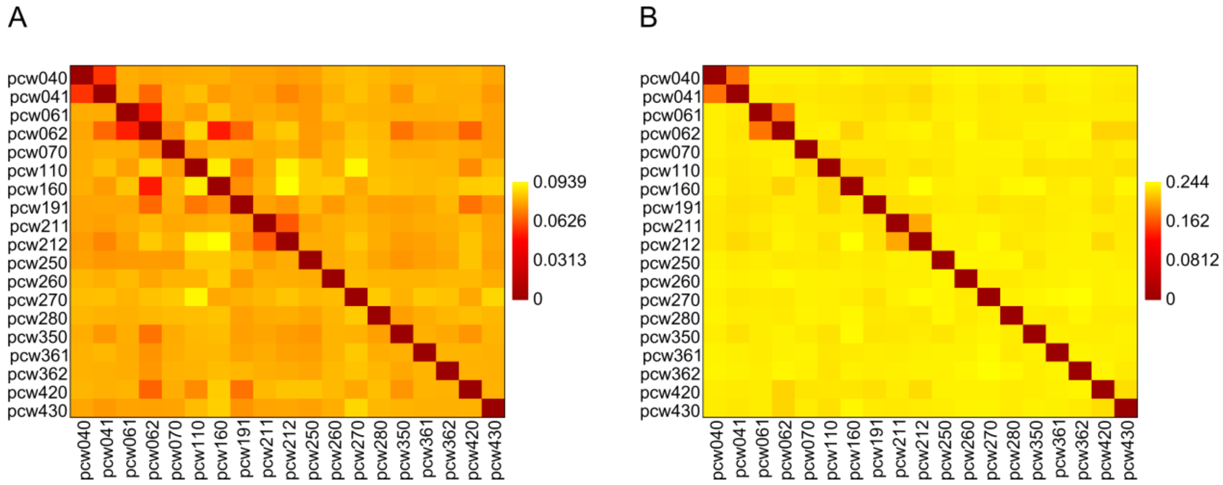

Figure SX17. Pair-wise nucleotide diversity between tested individuals merged with (A) Human Origins reference panel, (B) 1000 Genome Project reference panel.

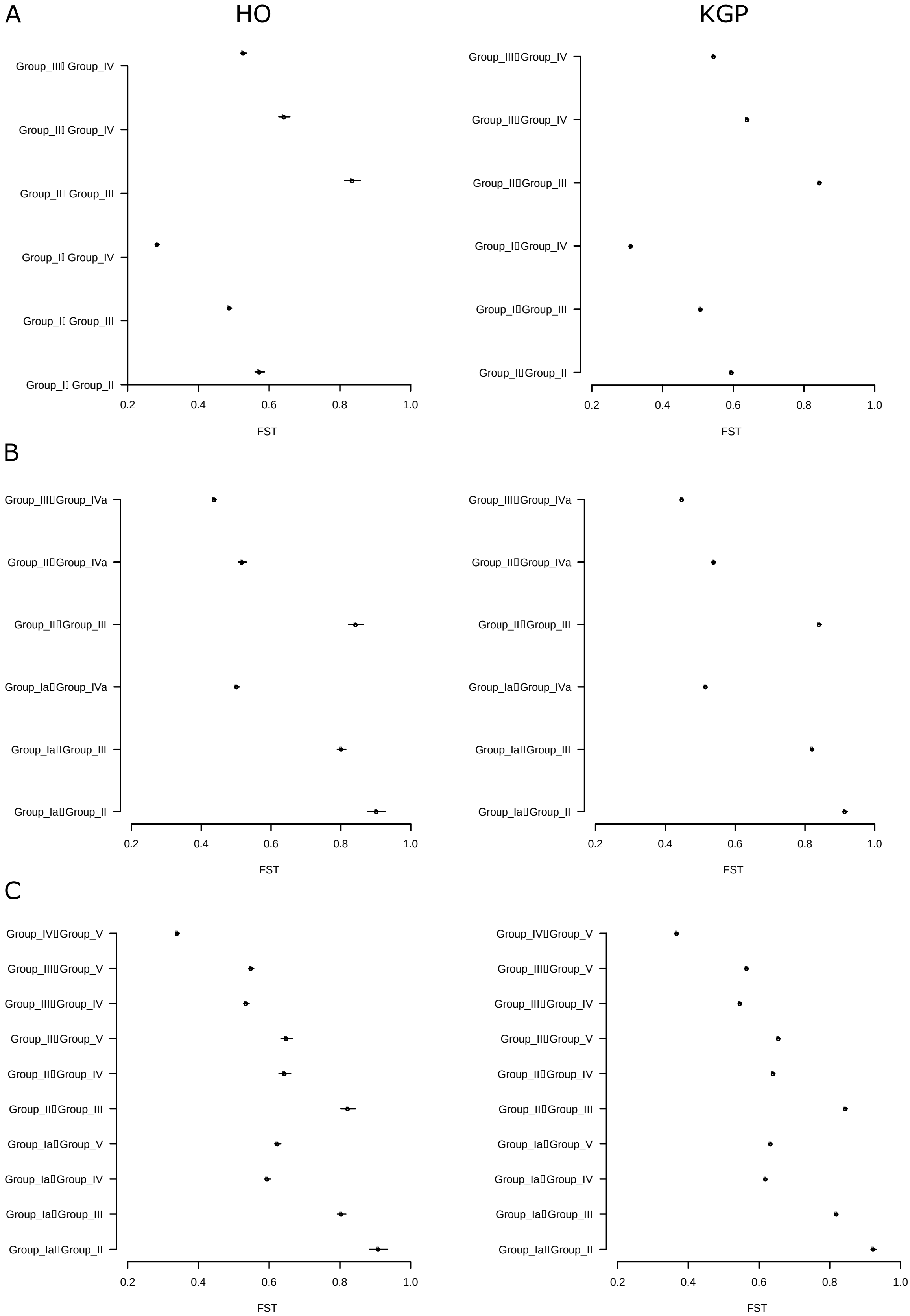

Figure SX18. FST distances calculated between alternative groupings of ancient individuals presented in this study using SNP panels merged both with Human Origins (left column) and

1000 Genomes Project (right column). A) the four groups as used in this paper (Groups I-IV); B) non-local individuals from the Rzeszów Foothills combined with individuals from the Sokal Ridge (Group Ia, Group II-III and Group IVa); C) five groups (Group Ia, Group II-V), where group V are non-local individuals from the Rzeszów Foothills.

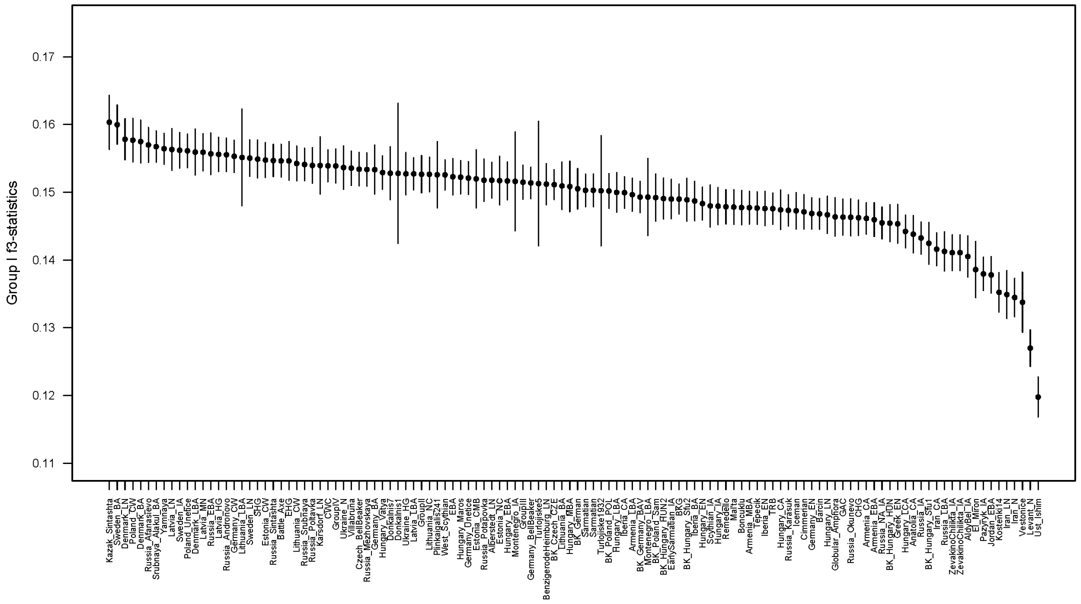

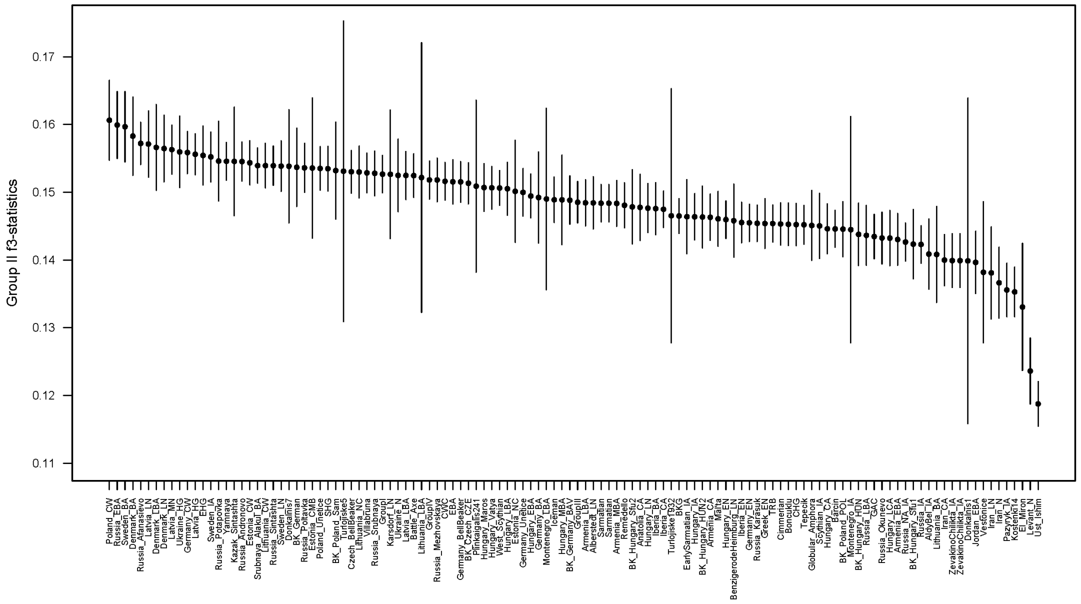

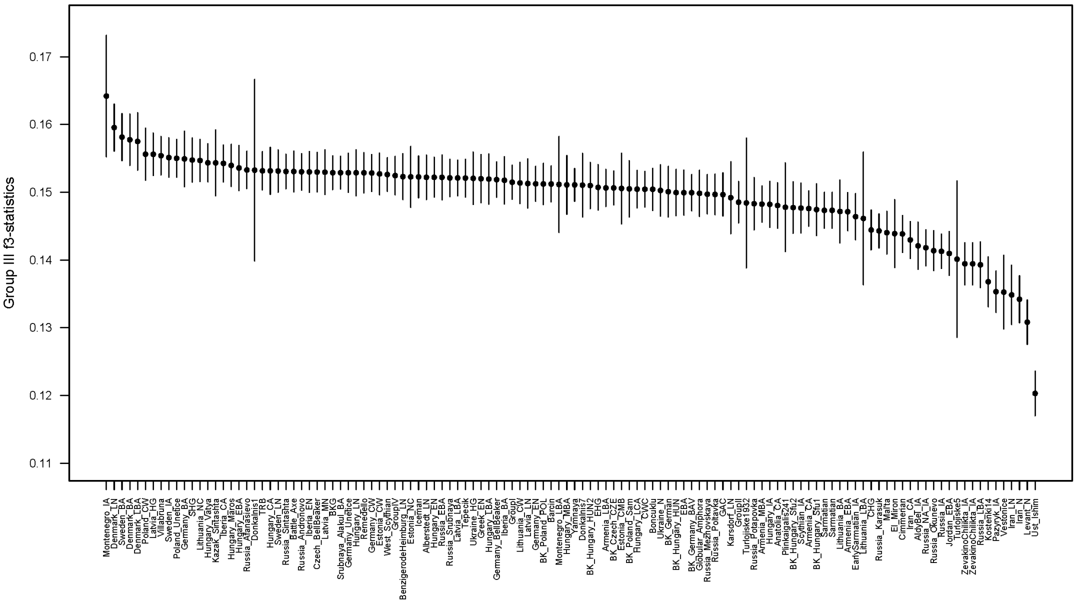

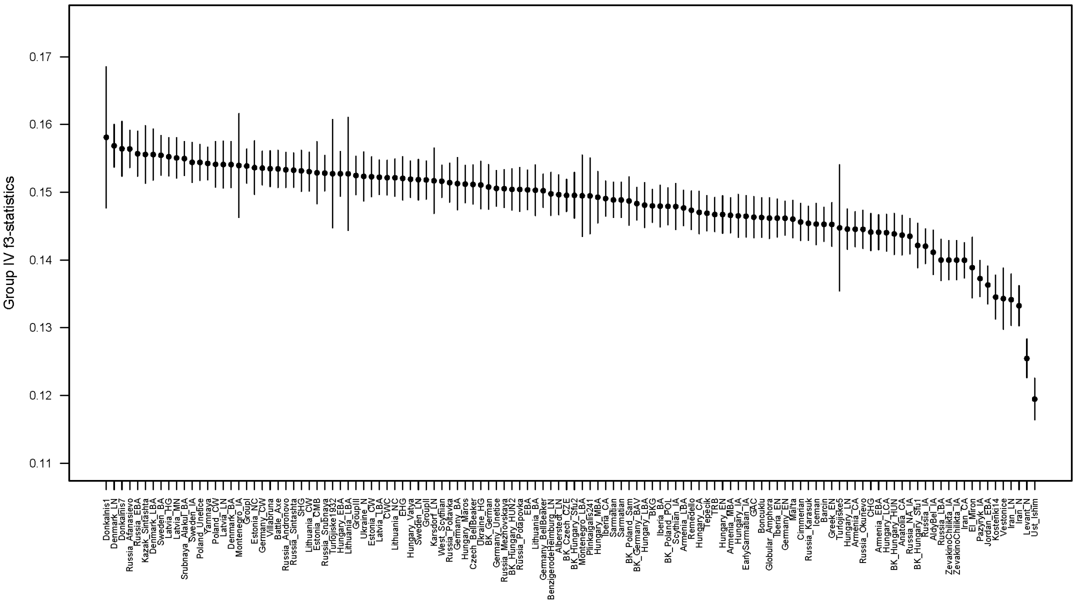

Figure SX19. Outgroup f_3-statistic between the tested individuals grouped according geographical and archaeological information and all ancient populations tested. Data in **Table SX14**.

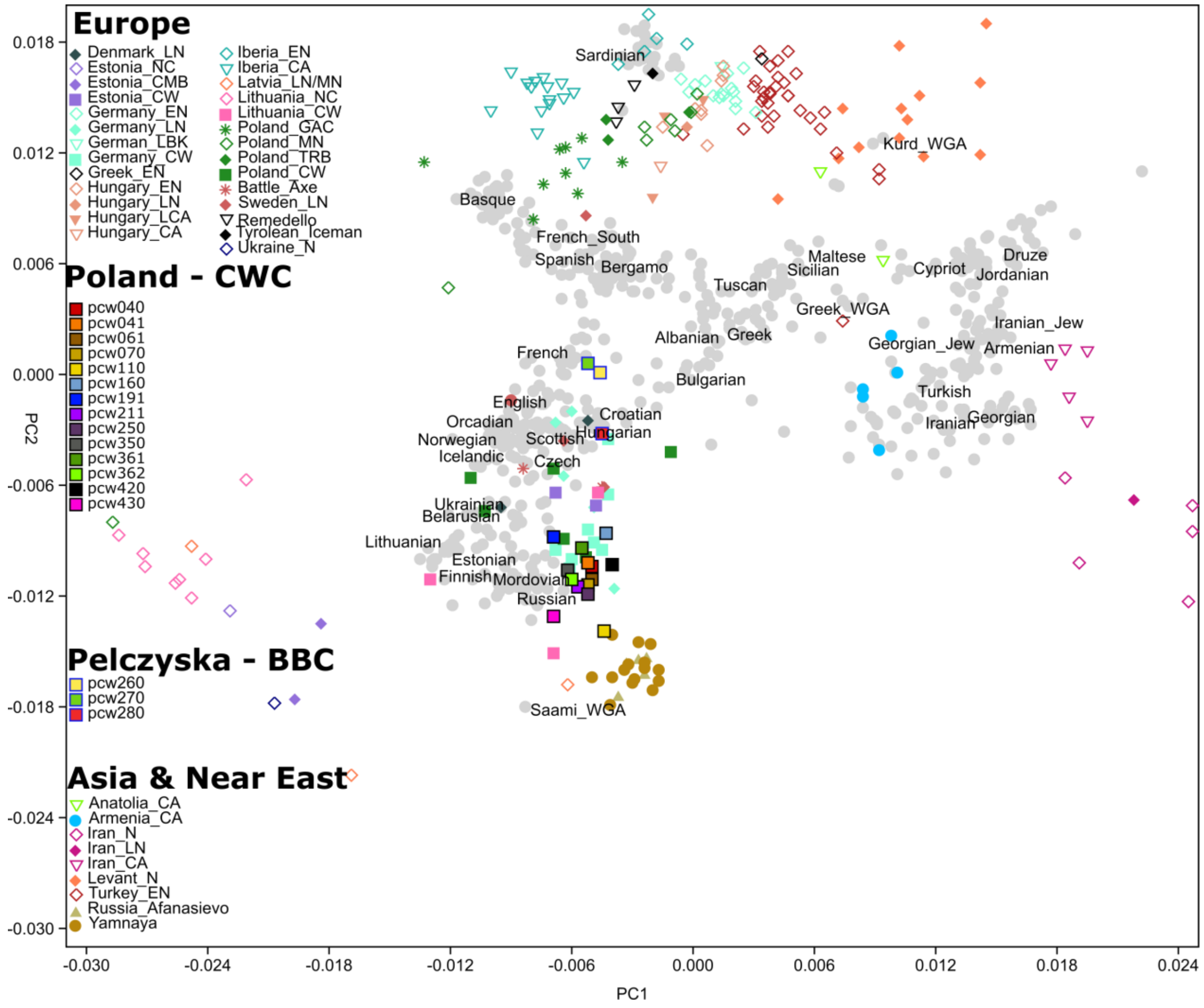

Figure SX20. PCA – Neolithic only with the studied samples marked individually.

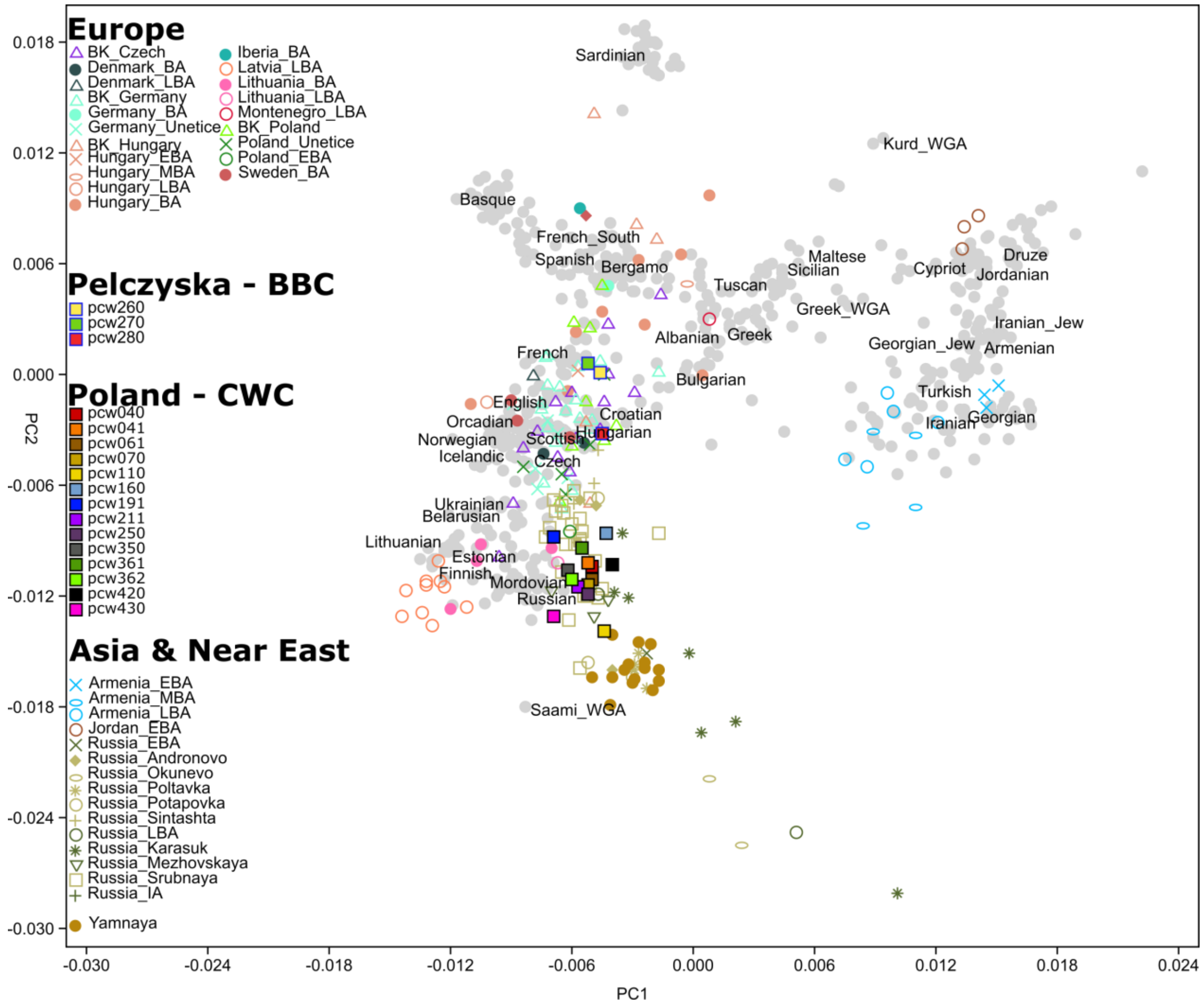

Figure SX21. PCA – Bronze Age only with the studied samples marked individually.

K=2

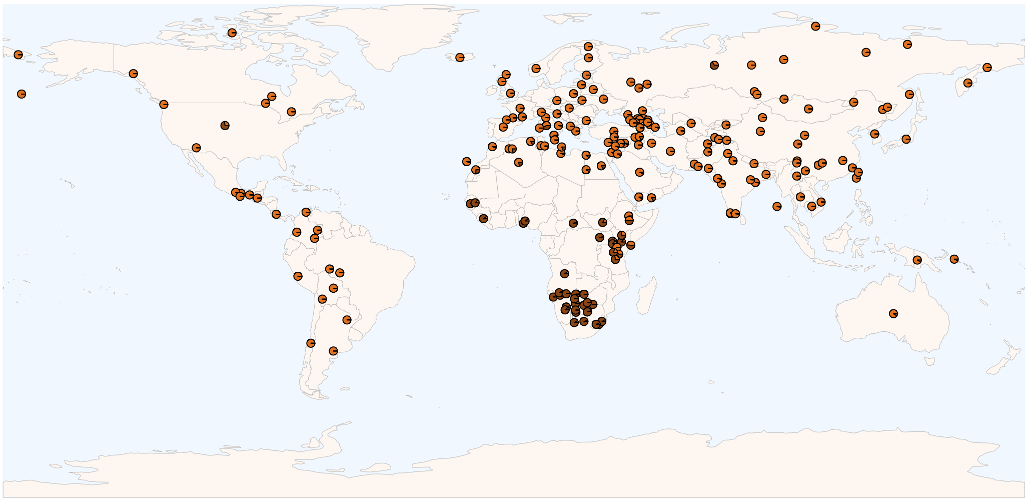

847

848 K=3

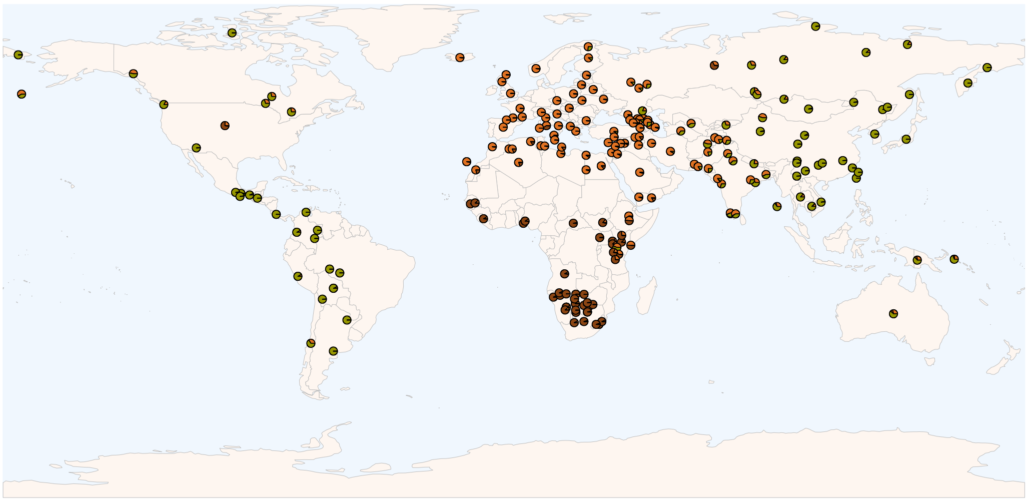

849

K=4

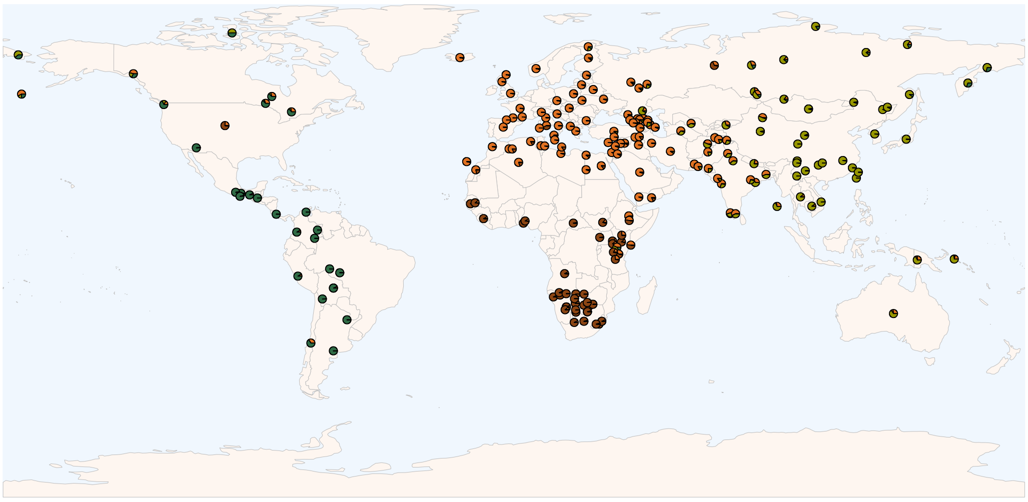

K=5

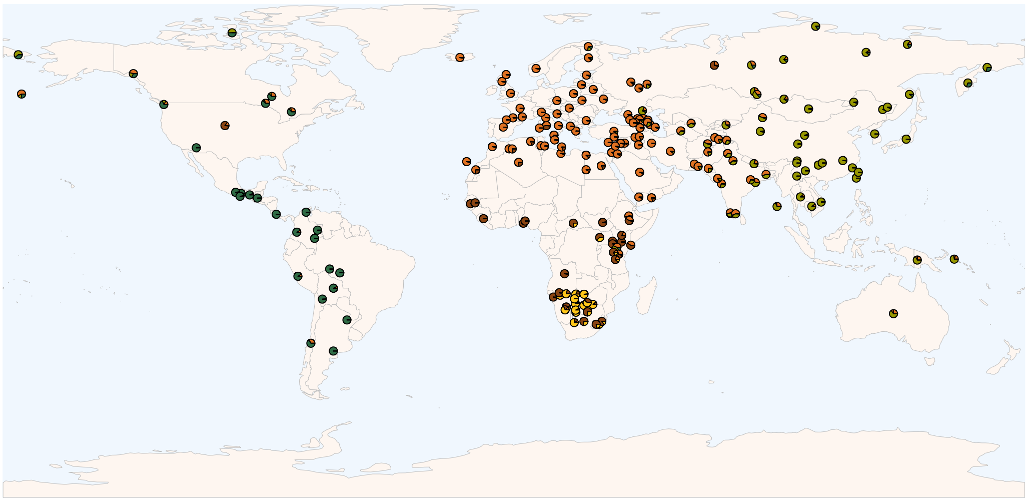

K=6

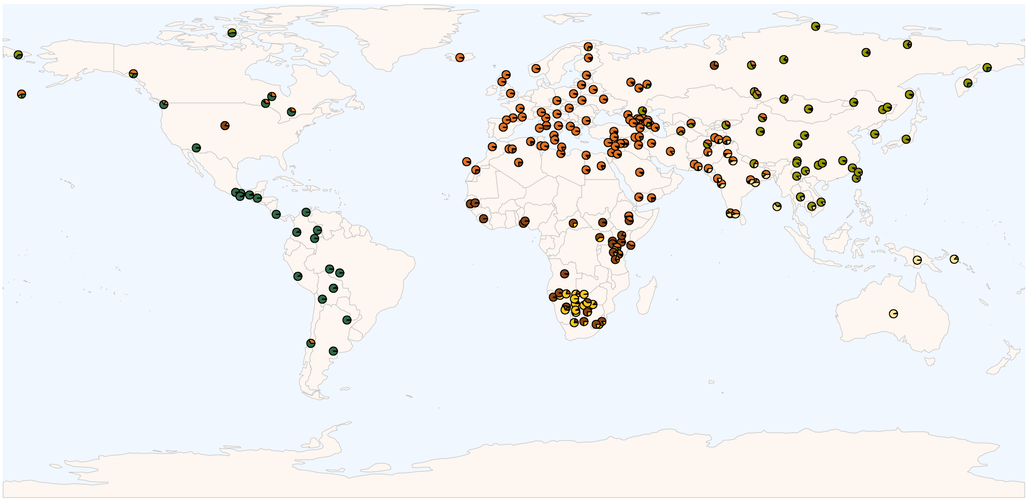

K=7

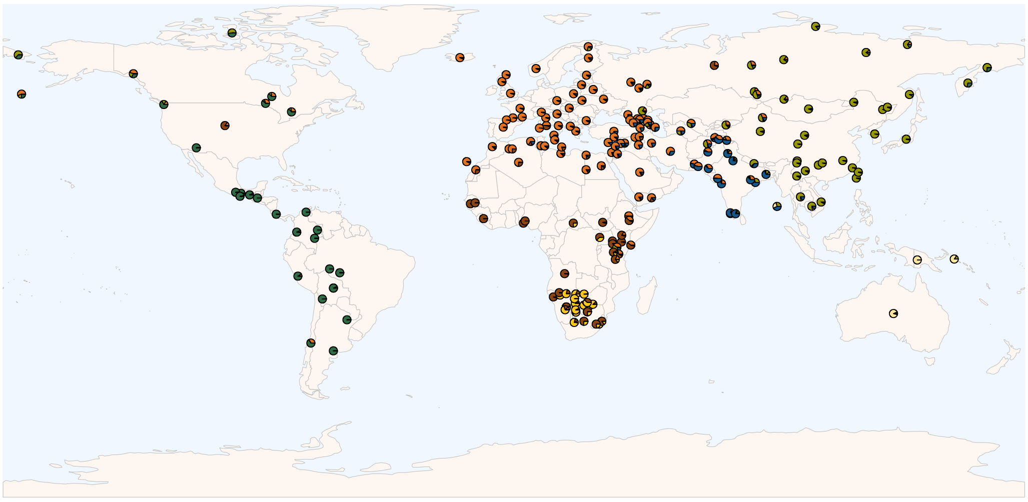

K=8

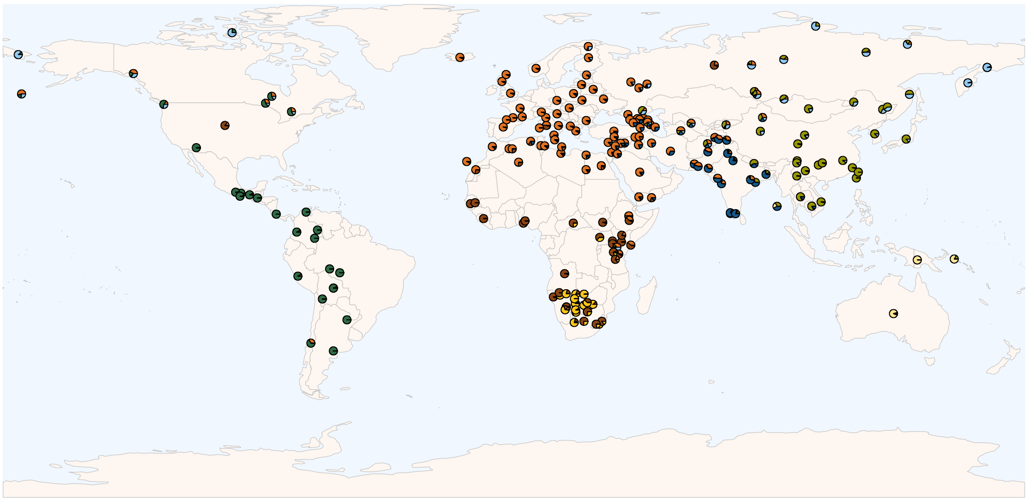

K=9

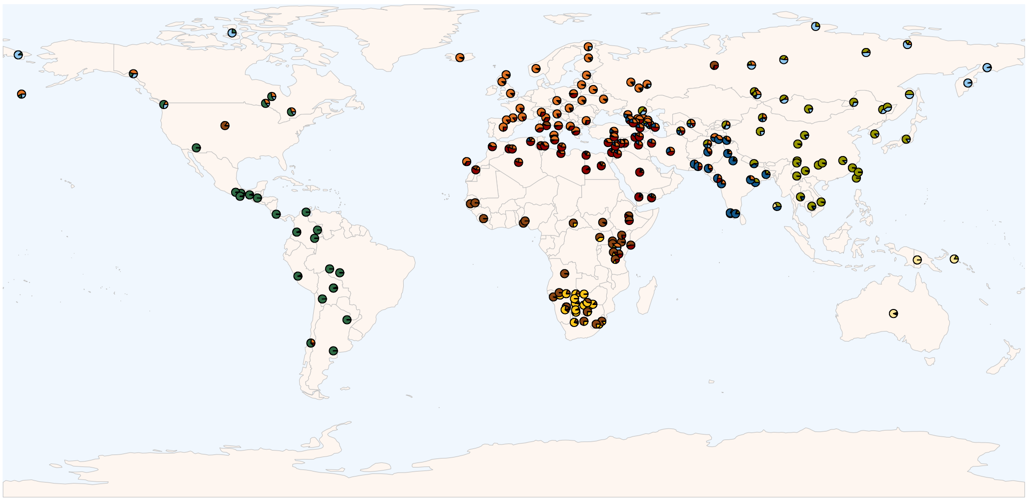

K=10

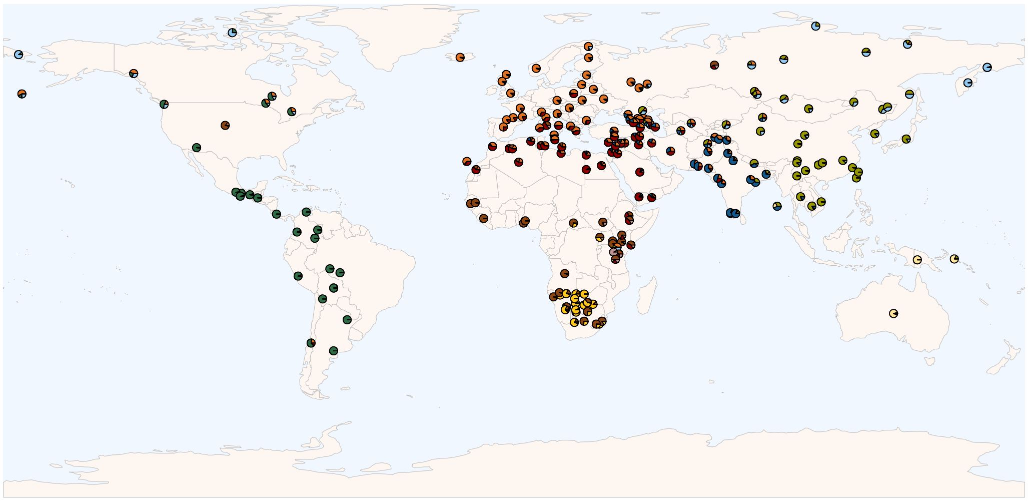

Figure SX22. Admixture analyses of modern populations at K=2 to K=10 results in individual maps.

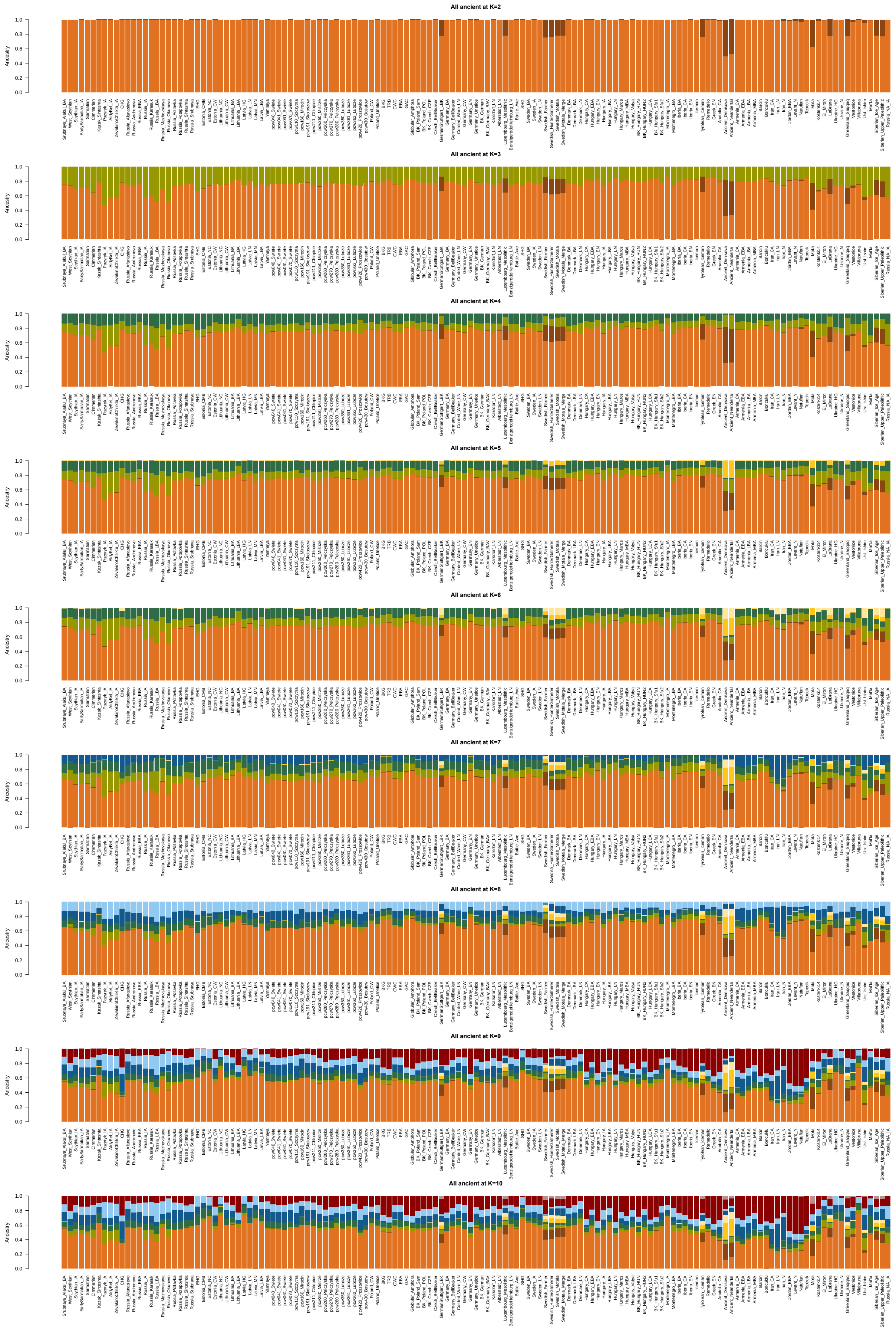

Figure SX23. Admixture analyses of ancient individuals with K=2 to K=10 results merged

References - Supplementary Information

1. Furholt, M., *Massive Migrations? The impact of recent aDNA studies on our view of third millennium Europe.* European Journal of Archaeology, 2018. **21**(2): p. 159-191.
2. Buchvaldek, M., *Zum gemeineuropäischen Horizont der Schnurkeramik.* Praehistorische Zeitschrift, 1986. **61**(2): p. 129-151.
3. Glob, P.V., *Studier over den jyske Enkeltgravskultur*. 1945: Gyldendal.
4. Struve, K.W., *Die Einzelgrabkultur in Schleswig-Holstein: und ihre Kontinentalen Beziehungen*. 1955: Wachholtz.
5. Buchvaldek, M. and C. Strahm, *Die kontinentaleuropäischen Gruppen der Kultur mit Schnurkeramik: Schnurkeramik-Symposium 1990*. Vol. 19. 1992: Karolinum.
6. Sulimirski, T., *Corded ware and globular amphorae north-east of the Carpathians*. 1968: Athlone Press.
7. Machnik, J., *Studia nad kultura, ceramiki sznurowej w Malopolsce*. 1966: Zaklad Narodowy im. Ossolinskich.
8. Machnik, J., *The Corded Ware culture and cultures from the turn of the Neolithic Age and the Bronze Age.* The Neolithic in Poland, 1970: p. 383-420.
9. Machnik, J., *Krąg kulturowy ceramiki sznurowej.* Prahistoria ziem polskich, 1979. **2**: p. 337-411.
10. Kośko, A., *From research into the issue of the development dependencies of the Corded Ware culture and Yamnaya culture In: S.* A Turning of Ages/Im Wandel der Zeiten. Jubilee Book Dedicated to Professor Jan Machnik on His 70th Anniversary, 2000: p. 337-346.
11. Kruk, J., *Przyczynki do badań nad eneolitem lessów Małopolski.* Archeologia Polski, 1974. **19**(2).
12. Kempisty, A., *Schylek neolitu i poczatek epoki brazu na Wyzynie Malopolskiej w świetle badan nad kopcami*. 1978: Wydawnictwa Uniwersytetu warszawskiego.
13. Włodarczak, P., *The traits of Early-Bronze Pontic cultures in the development of old upland Corded Ware (Małopolska groups) and Złota culture communities.* , in *Reception zones of „Early Bronze Age" Pontic culture traditions: Baltic Basin – Baltic and Black Sea drainage borderlands, 4/3 mil. to first half 2 mil. BC.*, A. Kośko, Editor. 2014. p. 7-52.
14. Kośko, A. and P. Włodarczak, *Final Eneolithic research inspirations: Subcarpathia borderlands between eastern and western Europe.* , in *Reception of Pontic culture traditions among the Final Eneolithic communities in the Subcarpathian region, IIIrd millennium BC.* , A. Kośko, P. Szczepanek, and P. Włodarczak, Editors. 2018. p. 259-290.
15. Juras, A., et al., *Mitochondrial genomes reveal an east to west cline of steppe ancestry in Corded Ware populations.* Scientific reports, 2018. **8**(1): p. 11603.
16. Włodarczak, P., *Chronometry of the Final Eneolithic cemeteries at Święte from the perspective of cultural relations among Lesser Poland, Podolia and the north-western Black Sea region.*, in *Reception of Pontic culture traditions among the Final Eneolithic communities in the Subcarpathian region, IIIrd millennium BC.* , A. Kośko, P. Szczepanek, and P. Włodarczak, Editors. 2018. p. 178-212.
17. Kruk, J. and J. Machnik, *Studia osadnicze nad neolitem wyżyn lessowych*. 1973: Ossolineum.
18. Włodarczak, P., *Kultura ceramiki sznurowej na Wyżynie małopolskiej. Kraków.* Polish with English summary, 2006.
19. Klein, L., *A brief validation of the migration hypothesis with respect to the origin of the Catacomb culture.* Soviet Anthropology and Archeology, 1963. **1**(4): p. 27-37.
20. Machnik, J. and A. Pilch, *Zaskakujące odkrycie zabytków kultury środkowodnieprzańskiej w Młodowie-Zakąciu koło Lubaczowa, w woj. przemyskim.* Sprawozdania archeologiczne, 1997. **49**: p. 143-170.
21. Machnik, J., et al., *Neolityczne kurhany na Grzędzie Sokalskiej w świetle badań archeologicznych w latach 1988-2006*. 2009: Polska Akademia Umiejętności.
22. Budziszewski, J., et al., *Kultura pucharów dzwonowatych na Wyżynie Małopolskiej*. 2010: Wydawnictwo Instytutu Archeologii i Etnologii Polskiej Akademii Nauk.

23. Budziszewski, J., E. Haduch, and P. Wlodarczak, *Bell Beaker culture in south-eastern Poland.* BAR INTERNATIONAL SERIES, 2003. **1155**: p. 155-182.
24. Haduch, E., *Szczątki kostne ludności kultury pucharów dzwonowatych z terenów Małopolski.* , in *Kultura pucharów dzwonowatych na Wyżynie Małopolskiej.*, J. Budziszewski and P. Włodarczak, Editors. 2010: Kraków.
25. Kruk, J., *Gospodarka w Polsce południowo-wschodniej w V-III tysiącleciu pne*. 1980: Zakad Nar Nauk.
26. Włodarczak, P., *Projekt badań chronologii absolutnej eneolitu i początków epoki brązu w Małopolsce.* Otázky neolitu a eneolitu našich krajın–2010. Nitra: Archeologický Ustav Slovenskej Akadé mie Vied, 2013: p. 373-387.
27. Olalde, I., et al., *The Beaker phenomenon and the genomic transformation of northwest Europe.* Nature, 2018. **555**(7695): p. 190.
28. Olszewski, A. and P. Włodarczak, *Święte 11: cemetery of Corded Ware culture.* , in *Reception of Pontic culture traditions among the Final Eneolithic communities in the Subcarpathian region, IIIrd millennium BC.* , A. Kośko, P. Szczepanek, and P. Włodarczak, Editors. 2018. p. 7-67.
29. Janczewski, P., P. Kraus, and P. Włodarczak, *Święte 15: cemetery of Corded Ware culture*, in *Reception of Pontic culture traditions among the Final Eneolithic communities in the Subcarpathian region, IIIrd millennium BC.* , A. Kośko, P. Szczepanek, and P. Włodarczak, Editors. 2018. p. 92-138.
30. Dobrakowska, T. and P. Włodarczak, *Święte 20: cemetery of Corded Ware culture.* , in *Reception of Pontic culture traditions among the Final Eneolithic communities in the Subcarpathian region, IIIrd millennium BC.* , A. Kośko, P. Szczepanek, and P. Włodarczak, Editors. 2018. p. 139-162.
31. Hozer, M., J. Machnik, and A. Bajda-Wesołowska, *Groby kultury ceramiki sznurowej i domniemane kultury mierzanowickiej w Szczytnej, pow. Jarosław – źródła, analiza wnioski.* , in *Nekropolie ludności kultury ceramiki sznurowej z III tysiąclecia przed Chr. w Szczytnej na Wysoczyźnie Kańczuckiej.*, P. Jarosz and J. Machnik, Editors. 2017, Fundacja Rzeszowskiego Ośrodka Archeologicznego. p. 7-130.
32. Rybicka, M., *Kultura pucharów lejkowatych na podkarpackich lessach.* Komentarz do badań „autostradowych" In: S. Czopek (ed.), Autostradą w przeszłość. Katalog wystawy. Rzeszów, 2011: p. 45-59.
33. Rybicka, M., M. Głowacz, and D. Król, *Datowanie radiowęglowe wielokulturowego cmentarzyska ze Skołoszowa, stanowisko 7, pow. jarosławski, woj. podkarpackie.*, in *Wielokulturowe cmentarzysko w Skołoszowie, stanowisko 7, pow. jarosławski w kontekście osadnictwa z neolitu i wczesnej epoki brązu we wschodniej części Podgórza Rzeszowskiego, Rzeszów.*, M. Rybicka, Editor. 2017. p. 113-142.
34. Jarosz, P., *Kultura ceramiki sznurowej na obszarze Karpat w dorzeczach górnej Wisły, Dniestru i Cisy.* , in *IAE PAN*. 2014.
35. Machnik, J., P. Jarosz, and M. Mazurek, *Groby ludności kultury ceramiki sznurowej w Mirocinie, pow. Przeworsk.*, in *Nekropola ludności kultury ceramiki sznurowej w Mirocinie na Wysoczyźnie Kańczuckiej.* , P. Jarosz, J. Machnik, and A. Szczepanek, Editors. 2019: Rzeszów. p. 7-139.
36. Mariotti, A., et al., *The abundance of natural nitrogen 15 in the organic matter of soils along an altitudinal gradient (Chablais, Haute Savoie, France).* Catena, 1980. **7**(1): p. 293-300.
37. Jarosz, P., et al., *Niche burials of the corded Ware culture at KraKóW-MistrzejoWice, site 85.* Sprawozdania Archeologiczne, 2015. **67**: p. 165-187.
38. Jarosz, P. and I. Mianowska, *The Corded Ware culture cemetery in Kraków-Mistrzejowice.* Sprawozdania Archeologiczne, 2011(63): p. 241-276.
39. Prokopowicz, J., *Nowe neolityczne stanowisko w Proszowicach.* Materiały Archeologiczne, 1966. **7**: p. 79-85.
40. Krauss, A., *Cmentarzysko kultury ceramiki sznurowej w Bosutowie, pow, Kraków.* Materiały Archeologiczne 1960(2): p. 61-67.

41. Rudnicki, M. and P. Włodarczak, *Graves of the Bell Beaker Culture at Pełczyska, Pińczów district.* . Sprawozdania Archeologiczne, 2010. **62**: p. 353-374.
42. Koman, W., *Wyniki ratowniczych badań kurhanu nr 2 kultury ceramiki sznurowej w Łubczu na stan. 25, pow. Tomaszów Lubelski.* Archeologia Polski Środkowowschodniej, 1999. **4**.
43. Jarosz, P. and P. Włodarczak, *Chronologia bezwzględna kultury ceramiki sznurowej w Polsce południowo-wschodniej oraz na Ukrainie.* Przegląd archeologiczny, 2007. **55**: p. 71-108.
44. Włodarczak, P., *Chronologia absolutna cmentarzysk późno-i schyłkowoneolitycznych na Wyżynie Lubelskiej.* Schyłek neolitu na Wyżynie Lubelskiej, Kraków, 2016: p. 537-548.
45. Ambrose, S.H. and L. Norr, *Experimental evidence for the relationship of the carbon isotope ratios of whole diet and dietary protein to those of bone collagen and carbonate*, in *Prehistoric human bone*. 1993, Springer. p. 1-37.
46. Włodarczak, P., J. Czebreszuk, and J. Müller, *The absolute chronology of the Corded Ware Culture in the south-eastern Poland.* The absolute chronology in central Europe during the 3rd millennium BC (Poznań/Bamberg/Rahden, Westf. 2001), 2001: p. 103-129.
47. Machnik, J., *Radiocarbon chronology of the Corded Ware Culture on Grzęda Sokalska. A Middle Dnieper traits perspective.* , in *The Foundations of Radiocarbon Chronology of Cultures between the Vistula and Dnieper: 3150-1850 BC.* 1999. p. 221-250.
48. Włodarczak, P., *Radiocarbon and dendrochronological dates of the Corded Ware culture.* Radiocarbon, 2009. **51**(2): p. 737-749.
49. Svyatko, S.V., et al., *New radiocarbon dates and a review of the chronology of prehistoric populations from the Minusinsk Basin, southern Siberia, Russia.* Radiocarbon, 2009. **51**(1): p. 243-273.
50. Górski, J., et al., *New evidence on the absolute chronology of the early Mierzanowice culture in south-eastern Poland.*, in *From copper to bronze. Cultural and social transformation at the turn of the 3rd/2nd millenia B.C. in Central Europe.*, M. Bartelheim, J. Peška, and J. Turek, Editors. 2013, Langenweissbach. p. 105-118.
51. Reimer, P.J., et al., *IntCal13 and Marine13 radiocarbon age calibration curves 0–50,000 years cal BP.* Radiocarbon, 2013. **55**(4): p. 1869-1887.
52. Włodarczak, P., *Battle-axes and beakers: The Final Eneolithic societies.* The Past Societies. Polish Lands from the First Evidence of Human Presence to the Early Middle Ages, 2017. **2**: p. 5500-2000.
53. White, T.D. and P.A. Folkens, *The human bone manual*. 2005: Elsevier.
54. Szczepanek, A., et al., *Understanding Final Neolithic communities in south-eastern Poland: New insights on diet and mobility from isotopic data.* PloS one, 2018. **13**(12): p. e0207748.
55. Belka, Z., et al., *Human mobility in the Final Eneolithic population of Swięte, south-eastern Poland: Evidence from strontium isotope data.* . Baltic-Pontic Studies, 2018. **23**.
56. Machnik, J., *Transfer Of Ideas And Cultural (Taxonomic) Traits Between The Vistula And Dnieper In The Late Neolithic. Archaeological Evidence On Subcarpathian Plateaus*, in *Reception zones of „Early Bronze Age" Pontic culture traditions: Baltic Basin – Baltic and Black Sea drainage borderlands, 4/3 mil. to first half 2 mil. BC.*, A. Kośko, Editor. 2014. p. 87-106.
57. McArthur, J.M., R. Howarth, and T. Bailey, *Strontium isotope stratigraphy: LOWESS version 3: best fit to the marine Sr-isotope curve for 0–509 Ma and accompanying look-up table for deriving numerical age.* The Journal of Geology, 2001. **109**(2): p. 155-170.
58. Werens, K., A. Szczepanek, and P. Jarosz, *Light stable isotope analysis of diet in Corded Ware culture communities: Święte, Poland,* , in *Reception of Pontic culture traditions among the Final Eneolithic communities in the Subcarpathian region, IIIrd millennium BC.* , A. Kośko, P. Szczepanek, and P. Włodarczak, Editors. 2018. p. 229-245.
